# Complexation of Green and Red Kaede Fluorescent Protein Chromophores by a Zwitterion to Probe Electrostatic and Induction Field Effects

**DOI:** 10.1021/acs.jpca.1c10628

**Published:** 2022-02-09

**Authors:** Eleanor K. Ashworth, Mark H. Stockett, Christina Kjær, Philip C. Bulman Page, Stephen R. Meech, Steen Brøndsted Nielsen, James N. Bull

**Affiliations:** † School of Chemistry, University of East Anglia, Norwich Research Park, Norwich NR4 7TJ, United Kingdom; ‡ Department of Physics, Stockholm University, SE-10691 Stockholm, Sweden; § Department of Physics and Astronomy, Aarhus University, Aarhus 8000, Denmark

## Abstract

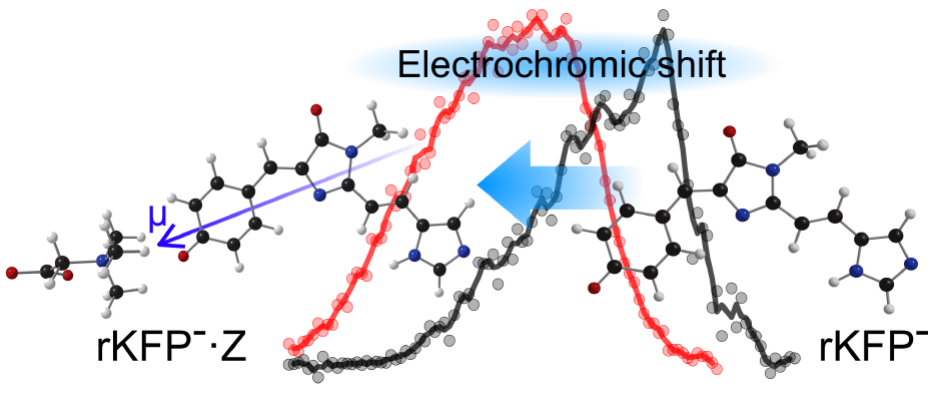

The photophysics of green fluorescent protein (GFP) and red Kaede fluorescent protein (rKFP) are defined by the intrinsic properties of the light-absorbing chromophore and its interaction with the protein binding pocket. This work deploys photodissociation action spectroscopy to probe the absorption profiles for a series of synthetic GFP and rKFP chromophores as the bare anions and as complexes with the betaine zwitterion, which is assumed as a model for dipole microsolvation. Electronic structure calculations and energy decomposition analysis using Symmetry-Adapted Perturbation Theory are used to characterize gas-phase structures and complex cohesion forces. The calculations reveal a preponderance for coordination of betaine to the phenoxide deprotonation site predominantly through electrostatic forces. Calculations using the STEOM-DLPNO-CCSD method are able to reproduce absolute and relative vertical excitation energies for the bare anions and anion–betaine complexes. On the other hand, treatment of the betaine molecule with a point-charge model, in which the charges are computed from some common electron density population analysis schemes, show that just electrostatic and point-charge induction interactions are unable to account for the betaine-induced spectral shift. The present methodology could be applied to investigate cluster forces and optical properties in other gas-phase ion–zwitterion complexes.

## Introduction

**Figure 1:**
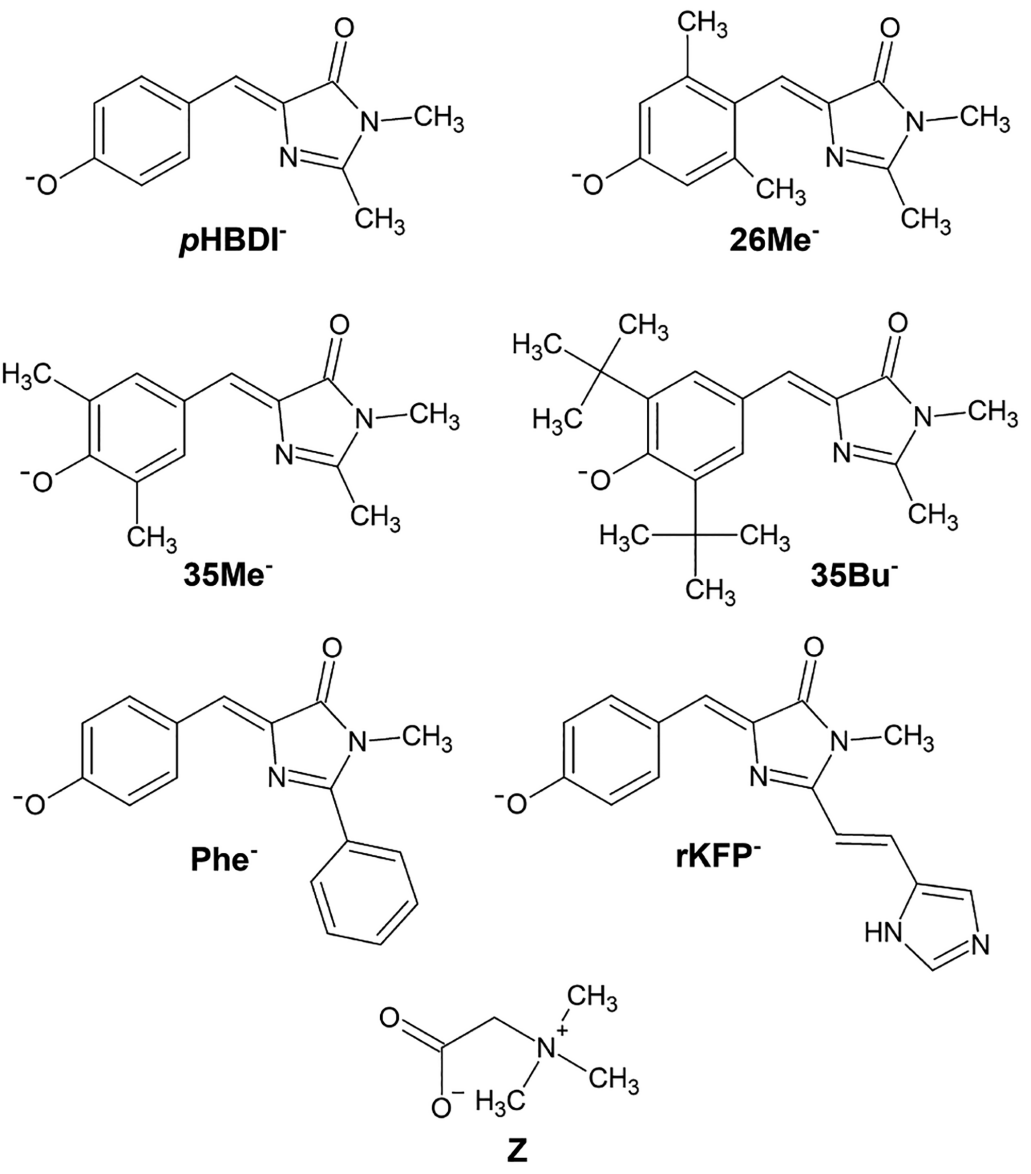
Molecular structures of the six target chromophore anions and betaine (Z). The presence of alkylation on the six-membered ring provides steric interactions around the deprotonation site. The negative charge in the chromophores is delocalized over both oxygen atoms.

The discovery of green fluorescent protein (GFP) from the *Aequorea victoria* jellyfish was a cornerstone in the “green revolution” of biological fluorescence imaging and the visualization of cellular processes.^1^ Although the *Aequorea victoria* jellyfish is an uncommon organism, the desirable optical properties of GFP and derivative fluorescent proteins and the ease with which they can be deployed as optical markers in biochemical systems has led to widespread use in photobiology.^2^ The optical absorption and emission properties of GFP are dictated by the S_1_ electronic state of a chromophore based on the deprotonated *p*-hydroxybenzylidene-2,3-dimethylimidazolinone (*p*HBDI^–^, Figure 1) unit that is situated within the β-barrel structure of the protein.^3^ The chromophore interacts with the protein binding pocket through a complex network of hydrogen bonds, involving amino acid residues and water molecules.^4^ The amalgamation of these interactions leads to the so-called electrochromatic shift; different electrostatic environments in the binding pocket for mutant proteins can tune the electrochromatic shift by up to 65 nm.^1,5^

The importance of electrostatic interactions in defining the electrochromatic shift and the detailed photophysics of GFP has been inferred through numerous experiments^6−9^ and large-scale calculations.^6,10−15^ Yet, it is interesting to note that the inherent (gas phase) S_1_ ←S_0_ absorption spectrum for *p*HBDI^–^ is only sightly shifted compared with the GFP absorption spectrum (≈37 meV at *T* ≈ 30 K^16^).^17−19^ It therefore has been argued that although the protein environment may have a determinative effect on the chromophore’s photophysics, including hindering isomerization and maximizing fluorescence quantum yield, there is no net perturbation to the Franck–Condon electronic structure in GFP leading to an electrochromatic shift.

The principal motivation for applying gas-phase action spectroscopy techniques to biochromophore molecules such as *p*HBDI^–^ is to characterize the inherent photophysics of the chromophore and thus directly inform on the total perturbation by the protein binding pocket. Furthermore, gas-phase studies potentially allow for straightforward comparison of experiment with theory and, where deviation exists, direction for refinement of theory. In a step toward probing the sensitivity of electronic transitions in gas-phase molecules to local electric field and intermolecular perturbations, Brøndsted Nielsen and co-workers^20^ proposed that information on the electronic character of the chromophore’s transitions and their susceptibility to electric field perturbations could be obtained through comparing gas-phase action spectra for bare ions with spectra for ion–betaine complexes. Briefly, betaine (*N*,*N*,*N*-trimethylglycine, Z in Figure 1) is a zwitterionic molecule possessing a substantial dipole moment (|μ| = 11.5–11.9 D)^21,22^ which exerts a dipolar field of 50–70 MV cm^–1^ at bonding separations in intermolecular complexes (several Angstroms). The crux of the betaine tagging strategy is that due to the zwitterionic charge distribution, the betaine molecule should preferentially coordinate with the charged site on a gas-phase ion, leading to a polarization and stabilization (lowering in energy) of the molecular orbitals interacting with the charged site. This effect should be most significant for ions with an asymmetric and highly polarizable charge distribution and can inform on the charge-transfer character of an electronic transition. For example, electronic transitions that have a substantial fraction of charge-transfer character are blue-shifted in gas-phase action spectra for the ion–betaine complex. This is because a charge-transfer transition involves migration of charge density away from the charge site.^23^ In contrast, symmetric molecules with low polarizability and/or those with electronic transitions that involve high local excitation character show only a small betaine-induced spectral shift (the red shift originating from symmetry breaking often cancels the blue shift due to induction forces). While the betaine tagging strategy certainly does not model all aspects of protein binding pockets, it provides an avenue for assessing microsolvation and perturbations by charged side chains to chromophore electronic structure.

This work reports a series of photodissociation action spectra as proxies for the absorption spectra of six *p*HBDI-based chromophores (Figure 1) and their betaine complexes. While the betaine tagging strategy has been previously applied to *p*HBDI^–^,^19^ there is no detailed analysis of betaine–anion binding interactions or calculations on the betaine-induced spectral shift. The choice of the alkylated *p*HBDI^–^ species shown in Figure 1 was because aqueous and alcoholic absorption spectroscopy measurements revealed substantial red shifts for the deprotonated anion, e.g., by 0.16 and 0.40 eV for 35Me^–^ and 35Bu^–^ compared with shifts for *p*HBDI^–^,^24^ and also because alkylation provides steric bulk around the deprotonation site and may direct betaine binding toward an alternative geometry. The origin for the red-shifted absorption of the alkylated anions in solution is unclear and was assumed inductive in origin,^25^ although it raises questions about the degree of charge-transfer character associated with the S_1_ ← S_0_ transition in isolated *p*HBDI^–^ molecules.^26^

The key questions that this study addresses are as follows: (i) How strongly do the *p*HBDI^–^ series bind with betaine and what is the nature of the binding forces? (ii) What is the influence of betaine binding on the action spectra? (iii) Can betaine-induced spectral shifts be reproduced by electronic structure calculations of anion–betaine complexes and/or treatment of betaine with point charges (electrostatics only)? (iv) Can we develop a simple computational protocol using standard electronic structure methods for analyzing anion–betaine complexes?

## Methods

### Experimental Details

Photodissociation action spectroscopy of the bare anions and anion–betaine complexes was performed using the Sep1 accelerator mass spectrometer.^27,28^ Electrosprayed anions were accumulated in an octupole ion trap that was emptied every 25 ms (40 Hz repetition rate). The ion bunches were accelerated to a kinetic energy of 50 keV and mass selected using a bending magnet. A nanosecond-pulsed laser system (EKSPLA NT342A, 20 Hz, unfocused) excited every second ion bunch midway along a 2.5 m linear flight region (10^–6^ Torr background pressure). Daughter ions were separated using an electrostatic energy analyzer situated after the laser-ion interaction region and detected with a channeltron. For the bare anions, loss of a methyl group accounted for more than 95% of the total photodissociation yield.^17,29−31^ For the anion–betaine complexes, loss of the betaine tag molecule was the only photodissociation channel under low laser fluence conditions (1–2 mJ pulse^–1^, ≈0.5 cm^2^); these conditions give rise to no more than a few percent of photodissociation yield. For a given wavelength, the difference in the number of counts between the “laser-on” and “laser-off” injections provided the photoinduced signal.

It is worth noting that photodissociation of the bare chromophores requires the absorption of two photons. Because the S_1_ ← S_0_ transition oscillator strengths are large (*f* > 0.8), the OPO fluence of 1–2 mJ pulse^–1^ was satisfactory to give good dissociation response across each action spectrum. Further discussion on photodissociation yield with OPO fluence for *p*HBDI^–^ is given in ref (19) and for rKFP^–^ is given in ref (31).

### Computational Details

#### Geometries

**Figure 2:**
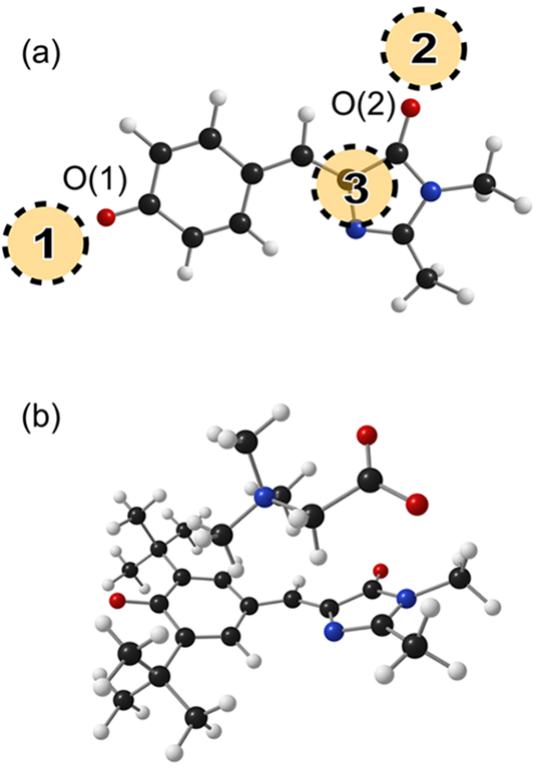
Anion–betaine complexes. (a) Major betaine binding sites (**1**, **2**, and **3**) shown with respect to *p*HBDI^–^. O(1) and O(2) indicate the phenolate and imidazolinone oxygen atoms, with the former being the deprotonation site. (b) Illustration of the 35Bu^–^·Z(**3**) complex involving side-on binding. Note that the HBDI backbone is slightly distorted due to complexation (allyl bridge dihredral angle is 19°).

Anion–betaine complex geometries were first optimized using the PM6 semiempirical Hamiltonian starting from a large number of test geometries, involving placing the betaine molecule at various positions around the chromophore core. The lowest energy geometries from these PM6 optimizations (and also the bare anions) were reoptimized at the ωB97X-D/aug-cc-pVDZ level of theory and confirmed to be geometrical minima through vibrational frequency analysis.^32−34^ The vibrational frequency calculations provided the zero-point energy (ZPE) corrections. The geometry optimizations revealed three betaine binding patterns shown in Figure 2a: **1** coordination to the phenoxide O(1) atom, **2** coordination to the imidazolinone O(2) atom, and **3** side-on coordination over the π-bonding system (see example in Figure 2b).

Using the ωB97X-D optimized geometries, we computed relative energies and adiabatic bond dissociation energies at the DLPNO-CCSD(T)/aug-cc-pVDZ level of theory with the TightPNO setting using ORCA 5.0.1.^35,36^ Benchmarking studies on the DLPNO-CCSD(T) method have shown the capacity to compute energies within ≈20 meV of CCSD(T) theory,^37−39^ although with a significant reduction in computational cost. Full CCSD(T) calculations on the present complexes are prohibitively expensive. Basis set superposition error (BSSE) corrections to the DLPNO-CCSD(T) calculations were included using the counterpoise method.^40^ It is relevant to note that diffuse basis functions are important for the correct description of the anionic chromophore and the anionic portion of the betaine molecule, and also to describe the dipole moment of betaine.

#### Anion–Betaine Binding Interactions

The intermolecular bonding interactions in the anion–betaine complexes were analyzed using symmetry-adapted perturbation theory, SAPT, as implemented in PSI4 version 1.3.2.^41^ SAPT is a well-established framework for decomposing complex interaction energies into physically meaningful interfragment terms.^42−44^ The SAPT framework uses perturbation theory to express the interaction energy, *E*_SAPT0_, as
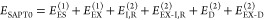
1where *E*_ES_^(1)^ and *E*_EX_^(1)^ are the first-order [superscript (1)] electrostatic term from two interacting charge densities and the exchange (Pauli repulsion) term, respectively. The remaining four terms are for second-order interactions [superscript (2)]: *E*_I,R_^(2)^ is electrostatic induction, i.e., the polarization of the molecular orbitals of one fragment (chromophore ion) by the electric field exerted by another fragment (betaine), *E*_EX-I,R_^(2)^ is the exchange contribution to the electrostatic induction energy, *E*_D_^(2)^ is the dispersion energy, i.e., London forces, and *E*_EX-D_^(2)^ is the exchange contribution to the dispersion energy. In physical chemistry, the *E*_ES_, *E*_I,R_^(2)^, and *E*_D_^(2)^ terms are usually divided into several subcomponents within classical point-charge models. In particular, *E*_ES_ includes interactions among permanent charges, dipoles, and quadrupoles (or generally multipoles). *E*_I,R_^(2)^ includes attractive interactions between a permanent multipole on one fragment and an induced multipole on another fragment. *E*_D_^(2)^, which contains London dispersion terms, are the weakest intermolecular forces and arise through temporary dipole attractive forces on both fragments, i.e., induced dipole–induced dipole attractions. SAPT calculations assumed the ωB97X-D optimized geometries and were mostly performed using the jun-cc-pVDZ basis set due to computational tractability and fortuitous cancellation of some errors such as the BSSE at the SAPT0 truncation level.^45^

#### Vertical Excitation Energies

Vertical excitation energies (VEEs) for the S_1_ ← S_0_ transition of the bare anions and anion–betaine complexes were computed at the DLPNO-STEOM-CCSD/aug-cc-pVDZ levels of theory.^46^ The choice of the DLPNO-STEOM-CCSD method over other wave function theories, such as CC2 or ADC(2), was because of good performance in several benchmarking studies on quantum chemical approaches for electrochromatic shifts.^47,48^ EOM-CCSD theory was not computationally feasible to apply to the betaine clusters.

To explore the electrostatic and point-charge induction contribution to the betaine-induced spectral shift, VEEs were computed assuming treatment of betaine with a point-charge model. In this model, betaine atoms in the complexes were replaced with point charges computed from one of three population schemes: (i) minimum basis set Mulliken populations (MBS),^49^ (ii) natural bond order (NBO) populations,^50^ and (iii) Hirshfeld-CM5 populations (CM5).^51^ The choice of minimum basis set Mulliken populations is because Mulliken population analysis with “large” basis sets, i.e., aug-cc-pVDZ, is known to be nonphysical.^49^ These three population schemes were considered in order to explore variation in computed atomic charges and because of ongoing controversy in which method provides the most robust set of point charges.

## Results and Discussion

### Photodissociation Action Spectra

**Figure 3:**
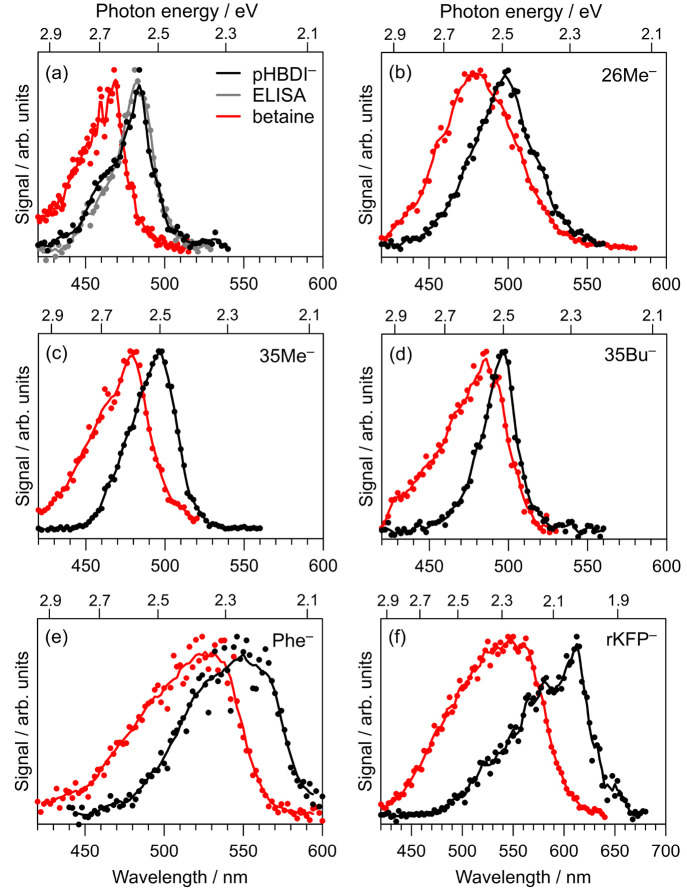
Photodissociation action spectra for bare ions (black) and anion–betaine complexes (red): (a) *p*HBDI^–^, (b) 26Me^–^, (c) 35Me^–^, (d) 35Bu^–^, (e) Phe^–^, (f) rKFP^–^. Action spectra for the bare anions were recorded by monitoring the loss of a methyl group (>95% of total photodissociation yield) and action spectra for the anion–betaine were recorded by monitoring loss of the betaine molecule. In (a), the gray data are a photoneutrals spectrum taken from ref (19) using the ELISA ion storage ring at Aarhus University. Solid lines are a moving average.

Photodissociation action spectra for the bare anions (black) and the betaine–anion complexes (red) are shown in Figure 3. Wavelengths of maximum response for each spectrum are given in Table 1. For *p*HBDI^–^, the gray ELISA spectrum in Figure 3a was taken from ref (19) and is a photoneutrals spectrum recorded in an ultrahigh vacuum ion storage ring. The close agreement of the present *p*HBDI^–^ spectrum recorded by monitoring methyl loss with the ELISA spectrum supports that the spectra recorded in this work should be good proxies for the S_1_ ← S_0_ absorption profiles. This conclusion is because the ELISA spectrum involved a long detection window (many microseconds) while the Sep1 detection window is tens of microseconds. Longer detection windows allow slower statistical dissociations, e.g., for longer wavelengths, to go to completion. Furthermore, both ELISA and Sep1 experiments involve dissociation in ultrahigh or high vacuum environment, which contrasts with many ion-trap-based experiments in which collisions may quench some dissociations and skew the action spectra.^52^

**Table 1:** Photodissociation Action Spectra Peak Wavelengths (λ in nm) for the Bare Anions and Anion–Betaine Complexes (Spectra in Figure 1), the Betaine-Induced Spectral Shift (Δ in eV), and the Calculated Vertical Detachment Energy (VDE in eV) at the DLPNO-CCSD(T)/aug-cc-pVDZ Level of Theory for the Bare Anion

species	λ_anion_	λ_betaine_	Δ^a^	VDE
*p*HBDI^–^	484 ± 2	468 ± 2	0.09	2.62
26Me^–^	498 ± 2	483 ± 2	0.08	2.69
35Me^–^	498 ± 2	476 ± 2	0.13	2.60
35Bu^–^	498 ± 2	486 ± 2	0.06	2.75
Phe^–^	552 ± 5	539 ± 5	0.05	2.82
rKFP^–^	615 ± 2	550 ± 5	0.24	2.96

a Uncertainty is approximately ±0.01 eV.

The photodissociation action spectra for the bare anions show a small red shift in peak wavelength with methylation (either 26Me^–^ or 35Me^–^) or *tert*-butylation of the core chromophore. Although to a lesser extent, this mirrors the trends seen in solution absorption spectra^24^ and is consistent with inductive effects from the alkyl functional groups. On the other hand, Phe^–^ and rKPF^–^ show significant red shifts compared with *p*HBDI^–^ results due to their extended conjugation. For these two chromophores, there is a concomitant increase in the calculated vertical detachment energy (VDE, Table 1). It is worth noting that gas-phase rKFP^–^ was recently investigated by some of the present authors using isomer-specific photodissociation action spectroscopy,^31^ where it was shown that three gas-phase forms may be generated using electrospray ionization (two deprotomers and one tautomer). Following on from that study, judicious choice of solvent and electrospray conditions in the present work allowed generation of a pure gas-phase ensemble of the phenoxide deprotomer shown in Figure 1. Finally, we note that the 26Me^–^·Z, 35Bu^–^·Z, and rKFP^–^·Z spectral profiles have different shapes (broadened) compared with that of the bare anion. For 26Me^–^·Z and 35Bu^–^·Z, this is consistent with twisting of the chromophore backbone due to betaine coordination (see next section). For rKFP^–^·Z, this may be due to a mixture of betaine binding sites in the gas-phase complexes.

Each of the chromophore anions may exist as *Z* and *E* geometric isomers with respect to the allyl bond linking the two rings in the core *p*HBDI unit. However, the spectra have been interpreted in terms of just the *Z* isomer for three reasons: (i) the synthesis and crystallization procedure exclusively yields the *Z* isomer, (ii) some of the present authors performed ion mobility experiments with *p*HBDI^–^ to show that electrospray ionization at *T* = 300 K produces only the *Z* isomer, with collisional activation of the gas-phase ions required to generate and kinetically trap the *E* isomer,^18^ and (iii) vibrational spectroscopy starting from electrosprayed ions at *T* = 300 K indicate only the *Z* isomer.^53^ The *E* isomer of *p*HBDI^–^ was calculated to lie 0.11 eV higher in energy than the *Z* isomer and the *Z–E* barrier was calculated at 1.26 eV, which is sufficiently large to prevent thermal isomerization at room temperature.^54^ For this work, we expect that only *Z* isomers are important because we used “gentle” ion source conditions to transfer nascent electrosprayed ions into high vacuum, i.e. minimal collisional activation, and because gentle ion source conditions are required to generate the anion–betaine complexes. Furthermore, betaine–anion complex binding energies (CBEs, see next paragraph) are lower than the *Z* → *E* isomerization barrier for *p*HBDI^–^ and presumably for the other chromophores.

The betaine-induced spectral shifts are summarized in Table 1. Compared with results for *p*HBDI^–^·Z, the shifts are smaller for 26Me^–^·Z, 35Bu^–^·Z, and Phe^–^·Z but are larger for 35Me^–^·Z and rKFP^–^·Z. The spectral shift for rKFP^–^·Z (Δ = 0.24 eV) is substantial and is similar to those previously reported for the oxyluciferin (≈0.20 eV) and *m*-nitrophenolate (≈0.30 eV) anions^20^ but less than half of that for protonated Schiff base retinal (≈0.60 eV).^55^ Both the *m*-nitrophenolate and the protonated Schiff base retinal in the gas phase are prototype systems known to undergo charge-transfer transitions; these trends suggest that the S_1_ ← S_0_ transition in rKFP^–^ has significant charge-transfer character.

The photodissociation action spectra can be compared with earlier aqueous and ethanol absorption spectra (Table 2). In aqueous solution, all six chromophores show blue spectral shifts (particularly 26Me^–^), although the 35Bu^–^ spectral shift is small. In ethanol, five of the six chromophores show blue spectral shifts, although to a lesser extent than in aqueous solution. The red spectral shift for 35Bu^–^ in ethanol relative to the gas phase (and small blue shift for aqueous) is consistent with the *tert*-butyl groups sterically encumbering solvation and therefore stabilization of the deprotonation site. It is also interesting to compare the betaine-induced spectral shifts with the solution absorption spectra. For example, the aqueous-induced shifts for *p*HBDI^–^ and 35Me^–^ are approximately 4-fold larger than the betaine-induced spectral shift, while the 26Me^–^ aqueous shift is around 7-fold larger (presumably because of geometry and charge-transfer changes). In contrast, for 35Bu^–^ the betaine-induced spectral shift is approximately 2-fold larger than the aqueous shift, attributed to the betaine binding pattern detailed in the following two subsections. The Phe^–^ and rKFP^–^ species have larger aqueous-induced spectral shifts than for *p*HBDI^–^, but similar to that for 26Me^–^. The larger spectral blue shift for *p*HBDI^–^ in solution (full coordination sphere) compared with the betaine complex is consistent with increased stabilization of the occupied frontier orbitals in solution; for example, it is well-known that biochromophores with phenoxide deprotonation show large solvation-induced shifts.^56^

**Table 2:** Photon Energies (in eV) at the Absorption Peak for the Bare Anions in Aqueous (*E*_aq_) and Ethanol (*E*_eth_) Solution^a^

species	*E*_aq_	*E*_eth_
*p*HBDI^–^	2.92^b^ (0.36)	2.81^b^ (0.24)
26Me^–^	3.02^b^ (0.53)	2.81^b^ (0.32)
35Me^–^	2.76^b^ (0.27)	2.60^b^ (0.12)
35Bu^–^	2.52^b^ (0.03)	2.44^b^ (−0.05)
Phe^–^	2.74 (0.49)	2.60 (0.15)
rKFP^–^	2.52^c^ (0.50)	2.34 (0.32)

a Values in parentheses are solvation-induced spectral shifts, i.e., relative to the bare anion.

b Reference (24).

c Reference (31).

### Anion–Betaine Binding

The optimized geometries for the anion–betaine complexes reveal three low energy coordination patterns, shown in Figure 2. Betaine binding site **1** has the positive end of the betaine molecule coordinated to the phenoxide oxygen atom O(1). Betaine binding site **2** has the positive end of the betaine molecule coordinated to the imidazolinone oxygen atom, O(2). In these geometries, the anion and betaine molecules are roughly in the same plane. For betaine binding site **3**, the betaine molecule is positioned side-on over the core *p*HBDI unit, with the positive end of the betaine molecule directed toward the phenoxide oxygen atom; see the example in Figure 2b. The calculated anion–betaine complex energies are given in Table 3. Aside from 26Me^–^·Z and 35Bu^–^·Z, betaine binding site **1** with coordination to the phenoxide deprotonation site corresponds to the lowest energy complex. For 26Me^–^·Z, the preference for betaine binding site **3** is linked to torsion of the allyl backbone due to intramolecular steric interactions by the methyl groups (see illustrations in the Supporting Information). For 35Bu^–^·Z, the preference for betaine binding site **3** is connected with steric interactions from the *tert*-butyl groups in site **1**; further details are given in the next section. We conclude that the *tert*-butylation provides sufficient steric bulk around the deprotonation site to direct coordination of betaine to an alternative site.

**Table 3:** Relative Anion–Betaine Complex Energy, *E* in meV, Complex Binding Energy (CBE in eV, i.e., Anion–Betaine → Anion + Betaine), and Vertical Detachment Energy (VDE in eV) of the Complex for Betaine Binding Site **1**/**2**/**3**^a^

	*E*	CBE	VDE
binding site	**1**/**2**/**3**	**1**/**2**/**3**	**1**/**2**/**3**
*p*HBDI^–^·Z	0/145/52	1.09/0.95/1.04	3.66/3.61/3.64
26Me^–^·Z	73/228/0	1.03/0.86/1.09	3.69/3.67/3.71
35Me^–^·Z	0/145/62	1.02/0.87/0.96	3.61/3.50/3.57
35Bu^–^·Z	83/104/0	0.88/0.85/0.96	3.81/3.60/3.77
Phe^–^·Z	0/166/10	0.95/0.78/0.94	3.71/3.64/3.71
rKFP^–^·Z	0/83/31	0.88/0.80/0.85	3.85/3.69/3.83

a All values are at the DLPNO-CCSD(T)/aug-cc-pVDZ level of theory and include BSSE correction.

The relative energies of the complexes given in Table 3 were sensitive to the incorporation of BSSE corrections. In particular, betaine binding site **3** was calculated as the lowest energy structure for each chromophore when BSSE corrections were neglected (true also at the ωB97X-D/aug-cc-pVDZ and MP2/aug-cc-pVDZ levels of theory). This is because of the increased variational freedom in the wave function due to betaine atomic orbital functions describing the π-system on the *p*HBDI unit, with BSSE corrections at ≈10% for betaine binding sites **1** and **2** and ≈30% for site **3**. As a check with a larger basis set, we computed the energies for the *p*HBDI^–^·Z complexes at the DLPNO-CCSD(T)/def2-TZVP level of theory. In this case, the BSSE corrections are much smaller and betaine binding site **1** is predicted to be 31 meV more stable than betaine binding site **3**. The conclusion is that betaine binding site **1** is the preferential site in the gas-phase for *p*HBDI^–^, 35Me^–^, Phe^–^, and rKFP^–^, although the electrosprayed ion beam at *T* = 300 K may have a minor contribution from binding site **3** complexes; see the Supporting Information.

Adiabatic anion–betaine complex binding energies (CBEs) and vertical detachment energies (VDEs), both including BSSE corrections, are given in Table 3. CBE values for sites **1** and **3** are 0.9–1.1 eV, which is roughly twice that for *p*HBDI^–^·H_2_O complexes.^57,58^ Computed VDE values are all roughly 1 eV higher for the complexes relative to the bare anions. An interesting outcome of complexation is that the S_1_ ← S_0_ absorption bands for each anion–betaine complex is situated below the respective detachment threshold, which will have important implications for the gas-phase excited state dynamics. For example, it is known that excitation of *p*HBDI^–^ at *T* = 300 K in the gas phase using a photon resonant with the peak in the S_1_ ← S_0_ band results in a competition among vibrational autodetachment, internal conversion, and isomerization on a subpicosecond time scale;^18,59,60^ however, the vibrational autodetachment channel will be unavailable in the betaine complex leading to a situation similar to that of the protein environment. Future time-resolved spectroscopy on the betaine complexes may prove insightful for exploring microperturbations to GFP chromophore photophysics.

### SAPT Analysis

The anion–betaine intermolecular binding interactions were analyzed using SAPT theory. The total SAPT0 energy and decomposed terms for each complex are summarized in Table 4. The total SAPT0 energies (*E*_SAPT0_) when expressed as CBE values indicate an overestimation of the complexation energy by ≈10% compared with the (adiabatic) DLPNO-CCSD(T) values in Table 4. The overestimation is, in significant part, because the SAPT framework calculates diabatic energies that neglects geometry relaxation effects for separated fragments. Inspection of the SAPT terms in Table 4 (usually as a pair of the binding interaction with exchange contribution) reveal some general trends:

- The electrostatic term dominates complex cohesion for betaine binding sites **1** and **2** for each of the six chromophores. While O(1) is the deprotonation site, electron delocalization through conjugation to O(2) likely means that the electrostatic term is dominated by charge–dipole interactions in both complex geometries (**1** and **2**). Taking into account Pauli repulsion, the electrostatic term accounts for 40–50% of the interaction energy (≈30% for 35Bu^–^·Z(**1**), discussed below), while induction and dispersion account for ≈30% and ≈20%, respectively.
- The predominant cohesion for betaine binding site **3** are dispersion forces, accounting for 50–60% of the complex cohesion. Although the electrostatic and induction terms are substantial for these complexes, they are largely countered by Pauli exchange due to the extensive overlap of the occupied molecular orbitals of the two fragments.
- For betaine binding sites **1** and **3**, increase of the basis set to jun-cc-pVTZ yielded only small changes in each term, indicating that the jun-cc-pVDZ basis set within the SAPT0 framework should provide a satisfactory description.

**Table 4:** Symmetry Adapted Perturbation Theory (SAPT) Analysis of the Anion–Betaine Complexes^a^

	*E*_ES_^(1)^	*E*_EX_^(1)^	*E*_I,R_^(2)^	*E*_EX-I,R_^(2)^	*E*_D_^(2)^	*E*_EX-D_^(2)^	*E*_SAPT0_	CBE
∗ *p*HBDI^–^·Z(**1**)	–131.6	72.3	–57.0	14.7	–28.6	5.3	–124.9	1.29
*p*HBDI^–^·Z(**1**)^b^	–128.5	72.9	–57.7	14.7	–36.2	6.3	–128.5	1.33
*p*HBDI^–^·Z(**1**)^c^	–128.3	72.8	–59.1	15.8	–37.8	6.6	–129.9	1.35
*p*HBDI^–^·Z(**2**)	–106.3	58.3	–40.2	10.9	–27.5	4.2	–100.4	1.04
*p*HBDI^–^·Z(**3**)	–114.4	92.8	–61.3	23.2	–71.4	8.9	–122.2	1.27
*p*HBDI^–^·Z(**3**)^c^	–111.9	92.7	–61.3	24.2	–87.0	10.6	–135.0	1.40
26Me^–^·Z(**1**)	–130.1	72.3	–57.5	18.7	–34.4	6.0	–125.9	1.30
26Me^–^·Z(**2**)	–107.7	63.1	–41.1	11.5	–32.8	4.7	–102.3	1.06
∗ 26Me^–^·Z(**3**)	–133.2	102.7	–66.0	24.6	–80.4	10.0	–142.3	1.48
∗ 35Me^–^·Z(**1**)	–131.2	78.0	–58.6	15.4	–34.5	5.9	–125.1	1.30
35Me^–^·Z(**1**)^c^	–127.9	78.4	–60.7	16.6	–44.6	7.2	–130.6	1.36
35Me^–^·Z(**2**)	–107.9	59.2	–41.1	11.1	–27.8	4.3	–102.3	1.06
35Me^–^·Z(**3**)	–116.4	95.4	–63.2	24.2	–73.9	9.2	–124.8	1.29
35Me^–^·Z(**3**)^c^	–113.7	95.3	–65.5	12.3	–89.8	10.9	–137.6	1.43
35Bu^–^·Z(**1**)	–114.6	81.0	–50.4	13.8	–49.3	6.3	–113.1	1.17
35Bu^–^·Z(**2**)	–104.3	57.2	–39.4	10.7	–27.9	4.2	–99.5	1.03
∗ 35Bu^–^·Z(**3**)	–124.2	110.3	–72.9	26.5	–89.0	10.7	–138.7	1.44
∗ Phe^–^·Z(**1**)	–126.2	69.1	–54.4	13.5	–22.8	5.1	–120.7	1.25
Phe^–^·Z(**2**)	–90.9	52.2	–37.5	11.7	–26.9	3.9	–87.4	0.91
Phe^–^·Z(**3**)	–110.2	91.4	–59.1	22.6	–73.9	9.1	–120.1	1.25
∗ rKFP^–^·Z(**1**)	–117.9	66.5	–52.1	16.5	–31.1	5.3	–112.7	1.17
rKFP^–^·Z(**2**)	–100.4	55.9	–38.4	10.4	–27.1	4.1	–95.6	0.99
rKFP^–^·Z(**3**)	–112.1	97.2	–64.8	24.0	–79.3	9.6	–125.4	1.30

∗ indicates the lowest energy complex.

a Contributions with superscript (1) are a first-order perturbation terms, and those with superscript (2) are second-order perturbation terms. Negative and positive terms indicate binding and repulsive interactions, respectively. Energies are in kJ mol^–1^ except for the complex binding energy (CBE), which is in eV.

b aug-cc-pVDZ basis set.

c jun-cc-pVTZ basis set.

It is interesting to examine the 35Bu^–^·Z species since one of the motivations to study the present series of chromophores was because alkylation around the deprotonation site, O(1), may sterically hinder betaine binding. In this case, betaine binding site **3** corresponds to the lowest energy complex, which contrasts with *p*HBDI^–^·Z and 35Me^–^·Z for which betaine binding site **1** is lower in energy. The SAPT0 terms for the *p*HBDI^–^·Z → 35Me^–^·Z → 35Bu^–^·Z alkylation series show only a small decrease in the electrostatic term for 35Me^–^·Z(**1**) compared with that for *p*HBDI^–^·Z(**1**), but a significant decrease for 35Bu^–^·Z(**1**) when the Pauli repulsion (steric) term is taken into account. On the other hand, there is an increase in the dispersion forces contribution for 35Bu^–^ complexes to ≈40% such that they dominate the complex cohesion forces.

In summary, betaine molecules are predominately coordinated to the phenoxide sites of anionic *p*HBDI chromophores through electrostatic forces. Introduction of steric hindrance from *tert*-butyl groups around the deprotonation site leads to alternative side-on complex binding in which dispersion forces are dominant.

### Anion–Betaine Spectral Shifts

Calculated vertical excitation wavelengths for the S_1_ ← S_0_ transition in the bare anions, anion–betaine complexes and point-charge complexes are given in Table 5. Before discussing these data, it should be outlined that the experimental betaine-induced spectral shifts correspond to averaged values for the complexes at *T* ≈ 300 K. This is because intermolecular complexes have “shallow” potential energy surfaces and are consequently highly fluxional. For example, the calculated average thermal energy for *p*HBDI^–^·Z(**1**) at *T* = 300 K from a harmonic partition function is 0.67 eV, which is nearly two-thirds of the CBE. For this reason, comparisons between calculations assuming only equilibrium geometries and experiments should focus on trends such as mean deviations rather than quantitative agreement.

**Table 5:** Calculated S_1_ ← S_0_ Transition Wavelengths (λ in nm; DLPNO-STEOM-CCSD/aug-cc-pVDZ Level of Theory) for the Bare Anions, Anion–Betaine Complexes, MBS, NBO, and CM5 Point-Charge Models of Betaine^a^

	bare anion	complex	MBS	NBO	CM5
binding site		**1**/**2**/**3**	**1**/**2**/**3**	**1**/**2**/**3**	**1**/**2**/**3**
*p*HBDI^–^	494 (0.05)	463 (0.03)/479/485	432/440/477	441/433/475	447/436/479
26Me^–^	499 (0.00)	479/444/490 (0.04)	480/477/476	487/449/468	481/458/448
35Me^–^	510 (0.06)	469 (0.04)/490/492	460/483/470	460/499/486	470/460/474
35Bu^–^	507 (0.04)	485/498/480 (0.03)	464/505/487	469/499/490	476/507/475
Phe^–^	564 (0.05)	551 (0.05)/567/533	538/506/531	542/548/527	548/544/536
rKFP^–^	608 (0.02)	568 (0.07)/568/541	573/576/551	568/575/549	585/582/554

a Values in parentheses are deviations in eV from the experimental data in Table 1. 1 eV = 96.49 kJ mol^–1^. This table is reproduced in eV in the Supporting Information.

Computed transition wavelengths for the bare anions (Table 5) are generally in good accord with the experimental data. Across the chromophore series, the mean deviation in terms of photon energy between bare anion calculation and experiment is 0.04 eV, with the largest deviation being for 35Me^–^ at 0.06 eV (comparable with Δ values in Table 1). Interestingly, for the lowest energy anion–betaine complexes, the mean deviation is the same at 0.04 eV, indicating that the STEOM-DLPNO-CCSD/aug-cc-pVDZ level of theory satisfactorily accounts for the betaine-induced spectral shift.

It is worth noting that the complexation of betaine causes geometric changes to the chromophore anions. For betaine binding sites **1** and **2**, the perturbation is small and calculated transition wavelengths for the bare chromophores anions at their complex geometries lead to spectral shifts of >0.01 eV. For 26Me^–^·Z(**3**) and 35Bu^–^·Z(**3**), which correspond to the lowest energy complexes for those chromophores, the side-on binding interaction causes an internal twisting of the allyl bridge. For 26Me^–^ (see illustrations in Supporting Information), the dihedral angle across the methylene bridge is 18° [bare anion] and 39° [26Me^–^·Z(**3**)]. For 35Bu^–^·Z(**3**), the dihedral angle is 19°. These twists alter the transition energy compared to the bare anion by ≈0.04 eV, accounting for much of the betaine-induced shift. Thus, for betaine binding site **3**, a substantial part of the betaine-induced shift results from distortion of the chromophore backbone. Again, the present calculations consider minimum energy geometries and do not account for gas-phase fluxionality in the experimental data.

In addition to the quantum mechanical treatment of betaine, vertical excitation wavelength calculations were performed using the STEOM-DLPNO-CCSD framework in which the betaine atoms were replaced with point charges computed using three common atomic population schemes (Table 5). Mean deviations of calculated values relative to experiment are 0.04 eV (MBS), 0.07 eV (NBO), and 0.10 eV (CM5). For comparison, the average difference in the action spectra between maxima for the bare anion and the anion–betaine complex is 0.11 eV. Thus, while the point-charge models correctly predict blue-shifted absorption, agreement with experiment is, on average, substantially worse (particularly CM5) than for quantum mechanical treatment of betaine. We conclude that more than just electrostatic and point-charge induction forces are necessary to account for experimental anion–betaine spectral shifts. Fortunately, the favorable computational scaling of the STEOM-DLPNO-CCSD method allows the methodology to be applied to substantially larger betaine complexes on conventional laboratory computing resources. For example, the 35Bu^–^·Z(3) complex with 69 atoms took ≈12 h to compute the first three excited states on a laboratory computer with dual Xeon E5-2680v2 CPUs and 256 GB RAM. The point-charge model calculations for the 35Bu^–^·Z complexes took ≈4 h.

## Conclusions

This work has reported photodissociation action spectra at *T* ≈ 300 K for bare anions and betaine complexes of six *p*HBDI-based molecules. In each case, betaine complexation leads to a blue shift of the action spectrum. For complexes in which betaine is coordinated to the phenoxide deprotonation site, electrostatic forces are primarily responsible for complex cohesion although induction and dispersion forces still have an important contribution. On the other hand, steric crowding around the deprotonation site and/or allyl backbone torsion result in a side-on complex geometry in which dispersion forces dominate cohesion of the complex. The anion–betaine spectral shifts are readily reproduced using the STEOM-DLPNO-CCSD method, although with the caveat that comparison with experiment is not always trivial because of fluxionality. A point-charge model does not satisfactorily account for the betaine-induced spectral shifts, consistent with the fact that induction and dispersion forces are important for complex cohesion. The methodology assumed in this work could be applied to characterize other anion–betaine complexes, or presumably more general ion-zwitterion complexes.

One of the original motivations for development of the betaine tagging strategy was to inform on the charge-transfer character of electronic transitions, provided there is a single and known betaine binding site for asymmetric target molecules. While the S_1_ ← S_0_ transition for *p*HBDI^–^ in the gas phase or in GFP is thought to involve some degree of charge-transfer character,^7,26,61^ deployment of a robust theoretical framework to charge-transfer properties in the chromophores/complexes considered in this work would be a useful direction for future work.

Future experiments will endeavor to apply time-resolved strategies such as femtosecond pump–probe photoelectron imaging across a similar series of *p*HBDI-based chromophores and complexes with molecules such as water, methanol, or betaine. Such experiment should inform on how complexation affects intrinsic photophysical properties including excited state lifetimes and photoisomerization propensity,^18,59^ as well as changes in vibrational autodetachment and internal conversion competitions due to the increased electron detachment thresholds in the complexes.

## Supporting Information Available

Solution absorption spectra of Phe^–^; 26Me^–^ and 26Me^–^·Z(**3**) geometries; tables of Boltzmann populations of the anion–betaine complexes with binding site, transition energies, and atomic coordinates (PDF)

## Notes

The data that support the findings of this study are available from the corresponding author upon reasonable request.
