# Supplementary material for: Complexation of Green and Red Kaede Fluorescent Protein Chromophores by a Zwitterion to Probe Electrostatic and Induction Field Effects
Source: J Phys Chem A. 2022 Feb 9;126(7):1158–67. doi: 10.1021/acs.jpca.1c10628 (PMC9628818; doi:10.1021/acs.jpca.1c10628)
Supplement: Supplementary file 1 — jp1c10628_si_001.pdf [file jp1c10628_si_001.pdf]

Supporting Information for 'Complexation of Green and Red Kaede Fluorescent Protein Chromophores by a Zwitterion to Probe Electrostatic and Induction Field Effects'

Eleanor K. Ashworth,*,† Mark H. Stockett,‡ Christina Kjær,¶ Philip C. Bulman Page,† Stephen R. Meech,† Steen Brøndsted Nielsen,¶ and James N. Bull†

†*School of Chemistry, Norwich Research Park, University of East Anglia, Norwich NR4 7TJ, United Kingdom*

‡*Department of Physics, Stockholm University, SE-10691 Stockholm, Sweden*

¶*Department of Physics and Astronomy, Aarhus University, Aarhus 8000, Denmark*

E-mail: james.bull@uea.ac.uk

Solution absorption spectra for Phe$^-$

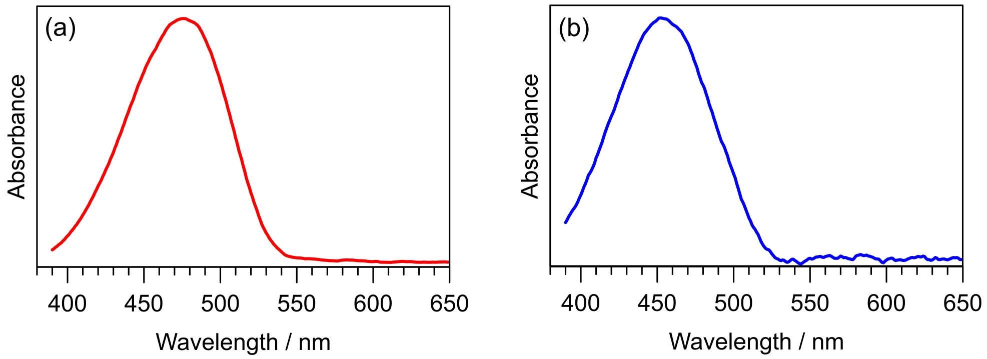

Figure S1: Absorption spectra of Phe$^-$ in (a) ethanol and (b) water. Both solutions contained a trace of NaOH to achieve deprotonation.

26Me$^-$ and 26Me$^-$·Z(3) geometries

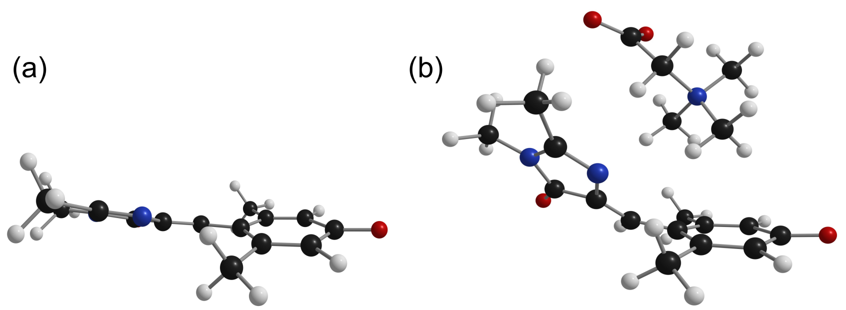

Figure S2: Illustrations of (a) 26Me$^-$ and (b) 26Me$^-$·Z(**3**) complex involving side-on binding, showing the internal twist about the core **p**HBDI bridge due to steric interactions from the methyl groups.

Boltzmann populations of the anion-betaine complexes with binding site

Table S1: Relative anion-betaine complex energy, ΔE, in kJ mol^{-1} and Boltzmann factor, N_i/N_j, relative to the lowest energy binding site complex for each anion-betaine species.

**Species**	ΔE	N_i/N_j
pHBDI$^-$·Z(**1**)	0.0	1.0
pHBDI$^-$·Z(**2**)	14.0	3.6×10^{-3}
pHBDI$^-$·Z(**3**)	5.0	1.3×10^{-1}
26Me$^-$·Z(**1**)	6.6	7.2×10^{-2}
26Me$^-$·Z(**2**)	22.2	1.4×10^{-4}
26Me$^-$·Z(**3**)	0.0	1.0
35Me$^-$·Z(**1**)	0.0	1.0
35Me$^-$·Z(**2**)	14.1	3.5×10^{-3}
35Me$^-$·Z(**3**)	5.8	9.9×10^{-2}
35Bu$^-$·Z(**1**)	8.2	3.7×10^{-2}
35Bu$^-$·Z(**2**)	10.4	1.5×10^{-2}
35Bu$^-$·Z(**3**)	0.0	1.0
Phe$^-$·Z(**1**)	0.0	1.0
Phe$^-$·Z(**2**)	16.4	1.4×10^{-3}
Phe$^-$·Z(**3**)	0.8	7.4×10^{-1}
rKFP$^-$·Z(**1**)	0.0	1.0
rKFP$^-$·Z(**2**)	8.1	3.9×10^{-2}
rKFP$^-$·Z(**3**)	2.7	3.4×10^{-1}

The use of Boltzmann populations to estimate the fraction of complexes with each betaine binding site requires one of two assumptions to be true: (i) betaine binding site scrambling with cascade to the lowest energy form occurs at the end of the electrospray process, i.e. just before final solvent evaporation; (ii) 'loose' transition states associated with gas-phase rearrangement are lower in energy than the complex binding energy but are also close to or above the average thermal energy of the ions at $T = 300$ K. For point (ii) assuming pHBDI$^-$·Z, we performed semi-empirical PM6 searches for transition states, identifying a **3**→**2** transition state roughly 0.5 eV higher in energy than species **2**. Because the average thermal energy for pHBDI$^-$·Z is ≈ 0.67 eV (see paper) and the complex binding energy is > 1 eV, statistical rearrangement may occur within a window of tens of microseconds before OPO probing. Higher-level calculations failed to converge. While we were unable to clearly identify a similar transition state for **3**→**1** rearrangement, PM6 calculations hinted at a pathway with a barrier situated 0.6–0.7 eV with respect to **3**. The loose transitions states for these rearrangements are situated below the complex binding energy in significant part due to the dispersion forces contributing to complex binding.

Table S2: Calculated $S_1 \leftarrow S_0$ transition energies (DLPNO-STEOM-CCSD/aug-cc-pVDZ level of theory) for the bare anions, anion-betaine complexes, MBS, NBO, and CM5 point charge models of betaine. Values in parentheses are deviations in eV from the experimental data in the paper. Note, this is a reproduction of Table 5 in the paper although with all data in units of eV.

Binding site	bare anion	complex **1/2/3**	MBS **1/2/3**	NBO **1/2/3**	CM5 **1/2/3**
*p*HBDI$^-$	2.51 (0.05)	2.68 (0.03) / 2.59 / 2.56	2.87 / 2.82 / 2.60	2.81 / 2.86 / 2.61	2.77 / 2.84 / 2.59
26Me$^-$	2.48 (0.00)	2.59 / 2.79 / 2.53 (0.04)	2.58 / 2.60 / 2.60	2.55 / 2.76 / 2.65	2.58 / 2.71 / 2.77
35Me$^-$	2.43 (0.06)	2.64 (0.04) / 2.53 / 2.52	2.70 / 2.57 / 2.64	2.70 / 2.48 / 2.55	2.64 / 2.70 / 2.62
35Bu$^-$	2.45 (0.04)	2.56 / 2.49 / 2.58 (0.03)	2.67 / 2.46 / 2.55	2.64 / 2.48 / 2.53	2.60 / 2.45 / 2.61
Phe$^-$	2.20 (0.05)	2.25 (0.05) / 2.19 / 2.33	2.30 / 2.45 / 2.34	2.29 / 2.26 / 2.35	2.26 / 2.28 / 2.31
rKFP$^-$	2.04 (0.02)	2.18 (0.07) / 2.18 / 2.29	2.16 / 2.15 / 2.25	2.18 / 2.16 / 2.26	2.12 / 2.13 / 2.24

Anion-betaine geometries

*p*HBDI^{-}·Z(1)

C	-0.7664916489	-0.6728958157	-0.0475345795
C	-0.1437576774	-1.9716748372	-0.0483319530
C	1.2231061098	-2.1113889940	-0.0377371747
C	2.1026610846	-0.9945843402	-0.0252251986
C	1.5037027378	0.2966479207	-0.0242442882
C	0.1391053105	0.4511184143	-0.0349087966
H	1.6585696250	-3.1144656158	-0.0386891199
H	2.1552425537	1.1692934255	-0.0147441346
C	3.5072572428	-1.2230828661	-0.0144190486
H	3.8176328406	-2.2727834166	-0.0171803430
O	-2.0264388658	-0.5252357798	-0.0571306408
C	4.5700658949	-0.3560084195	-0.0007531087
C	5.9596783685	-0.8156347562	0.0088469408
C	5.7761306828	1.4350721892	0.0196252259
N	4.5405290082	1.0512373995	0.0066696709
N	6.6831926458	0.3859362579	0.0217382229
C	6.2327792053	2.8537205770	0.0314016882
H	6.8514112203	3.0799973356	-0.8498716487
H	5.3527033846	3.5034183034	0.0281504963
H	6.8373896607	3.0698077645	0.9248669324
O	6.4743442352	-1.9318019550	0.0072067505
C	8.1200709187	0.4591121951	0.0351036700
H	8.5052433573	0.9805326086	-0.8530625735
H	8.4891910709	0.9694873450	0.9364163176
H	8.4896609012	-0.5727314122	0.0320908657
H	-0.7979416558	-2.8445819450	-0.0576348827
H	-0.3032065537	1.4494188403	-0.0340938440
C	-6.5649841352	1.0958250105	0.0114180812
H	-6.6131905311	1.7636272754	0.8798482980
H	-6.6586051699	1.7061001614	-0.8947966088
C	-7.7882342428	0.1188463253	0.0746590105
O	-8.8570282991	0.7522796032	0.0778907768
O	-7.5617235302	-1.1096057235	0.1127470193
C	-4.9814024721	-0.3709249046	-1.1998008433
H	-5.7225690555	-1.1709715977	-1.1328693120
H	-5.1563522020	0.2358678912	-2.0953967070
H	-3.9499410893	-0.7397834178	-1.1752416056
C	-4.9178334487	-0.2894330885	1.2339108639
H	-3.8891206311	-0.6627126617	1.1787475006
H	-5.0439510782	0.3768011812	2.0947803662
H	-5.6617694594	-1.0893320406	1.2605815930
C	-4.1787148624	1.6214204984	-0.0682470949
H	-3.1742792458	1.1737567302	-0.0770782534
H	-4.3642327297	2.2004904899	-0.9792850771
H	-4.3207756881	2.2629038940	0.8081414850
N	-5.1734607868	0.5109259506	-0.0052319387

pHBDI$^-$·Z(2)

C	5.57506964	-2.26973618	-0.00028535
C	4.27445307	-2.93405613	-0.00021193
C	3.11085808	-2.22431466	-0.00023353
C	3.08080558	-0.78933575	-0.00032317
C	4.35048159	-0.11580761	-0.00039803
C	5.52428520	-0.80850102	-0.00037967
H	2.15488123	-2.75693711	-0.00017976
H	4.35021623	0.97352935	-0.00046735
C	1.84498979	-0.13592998	-0.00034246
H	0.96838395	-0.79191391	-0.00028744
O	6.64741764	-2.90080607	-0.00027188
C	1.50096698	1.21353030	-0.00043819
C	0.13604185	1.67032609	-0.00050402
C	1.61024332	3.36936643	-0.00062684
N	2.36688893	2.31720050	-0.00055276
N	0.25791429	3.05844933	-0.00054331
C	2.08804406	4.78081404	-0.00074335
H	1.73283073	5.32604457	0.88686618
H	3.18207619	4.77608186	-0.00075050
H	1.73281977	5.32589847	-0.88843828
O	-0.96415308	1.07549858	-0.00050997
C	-0.85556686	3.97089509	-0.00054660
H	-0.84965950	4.60998378	0.89362869
H	-0.84969485	4.60991317	-0.89477159
H	-1.76850962	3.36527004	-0.00050240
H	4.27514763	-4.02509038	-0.00014127
H	6.48336665	-0.28772908	-0.00043500
C	-4.55225931	-2.22709429	0.00112918
H	-4.38212166	-2.84666051	-0.88726560
H	-4.38157999	-2.84645045	0.88956691
C	-6.04913905	-1.76061449	0.00152449
O	-6.81477632	-2.73668363	0.00185442
O	-6.28187513	-0.53162628	0.00147737
C	-3.58611199	-0.30601160	1.22048769
H	-4.57505058	0.15845349	1.19857121
H	-3.48902541	-0.95460571	2.09796180
H	-2.76986978	0.42293285	1.18790752
C	-3.58680972	-0.30633733	-1.21930477
H	-2.77054434	0.42260952	-1.18737958
H	-3.49022814	-0.95516704	-2.09665992
H	-4.57572886	0.15814258	-1.19694963
C	-2.13774382	-1.82426204	0.00037356
H	-1.37474653	-1.03695953	0.00001182
H	-2.05473207	-2.44971874	0.89505154
H	-2.05527896	-2.45000309	-0.89415635
N	-3.47611484	-1.16650543	0.00067510

*p*HBDI$^-$·Z(3)

C	4.88928544	0.29299808	0.45447030
C	4.60996401	0.00617749	-0.94552131
C	3.45981520	-0.62531203	-1.33736007
C	2.45094130	-1.02986834	-0.41115948
C	2.72178449	-0.79512704	0.97388479
C	3.86839366	-0.17257150	1.38687895
C	1.22759232	-1.53726655	-0.90835846
H	1.18346761	-1.69822372	-1.99068116
O	5.92043448	0.88950275	0.82953537
C	0.01992768	-1.78090270	-0.29023036
C	-1.19026388	-2.09117172	-1.04792474
C	-1.59701798	-1.76072452	1.14485037
N	-0.30925400	-1.59627206	1.06659774
N	-2.18938761	-2.05461525	-0.06925876
C	-2.41619834	-1.59799430	2.37741405
H	-3.00228093	-2.50453014	2.58597138
H	-1.74768383	-1.39933103	3.22072091
H	-3.11703884	-0.75791368	2.24750776
O	-1.38859428	-2.29283410	-2.24703659
C	-3.60626545	-2.13648494	-0.34410141
H	-4.05669261	-3.00396418	0.15837435
H	-4.10921931	-1.21449205	-0.02244423
H	-3.71216381	-2.25735468	-1.42741979
C	-1.70585907	1.66518427	0.70230088
H	-1.45852287	0.62036164	0.90062819
H	-1.60737017	2.22237605	1.64201430
C	-3.18038395	1.77120233	0.20703348
O	-3.94479469	1.07660306	0.90382775
O	-3.42412626	2.51793110	-0.76487594
C	-0.73164179	3.64835828	-0.40218205
H	-1.72071556	3.84728457	-0.82511706
H	-0.62823108	4.12741403	0.57723661
H	0.07640189	3.97882322	-1.06396502
C	-0.70972171	1.49407599	-1.54530603
H	0.12664729	1.83494808	-2.16406471
H	-0.62897037	0.41625429	-1.38474278
H	-1.67339508	1.75461588	-1.98730043
C	0.69845439	1.84381913	0.40859775
H	1.51065788	2.12468225	-0.26967008
H	0.78744179	2.39046560	1.35206355
H	0.73739092	0.77020610	0.59960781
N	-0.61609912	2.17559040	-0.21671670
H	4.06130648	-0.00402694	2.44806391
H	5.36041403	0.31493816	-1.67526587
H	3.28714063	-0.81533248	-2.40104597
H	1.97840927	-1.11609667	1.70384765

26Me^{-}·Z(1)

C	1.08540853	1.79823780	-0.22939318
C	0.12057978	2.74387417	0.25985743
C	-1.23350750	2.50620431	0.22456002
C	-1.75502219	1.25914037	-0.26980847
C	-0.82136583	0.32532305	-0.82733003
C	0.52915438	0.60656851	-0.80882803
C	-3.17034636	1.06838661	-0.19681790
H	-3.76345665	1.98600439	-0.16399008
O	2.33557740	2.00485614	-0.16698092
C	-3.99760414	-0.02238616	-0.10113911
C	-5.45646240	0.14754066	-0.05258411
C	-4.81281977	-1.99025554	0.25980917
N	-3.68457790	-1.37872686	0.10294999
N	-5.91727032	-1.15462869	0.17405442
C	-4.96571258	-3.44969021	0.52024482
H	-5.56013738	-3.93393020	-0.26874036
H	-3.97162557	-3.90500250	0.55083892
H	-5.47554659	-3.63163518	1.47792430
O	-6.18523097	1.13006015	-0.16358429
C	-7.30639441	-1.50716884	0.30377004
H	-7.61367605	-2.22429788	-0.47083828
H	-7.52217850	-1.93610488	1.29286071
H	-7.88100983	-0.58177689	0.18285266
H	0.50771676	3.67849393	0.66962049
H	1.22697302	-0.08851049	-1.28169562
C	5.72778713	-1.42499600	0.61126317
H	5.82771135	-1.48965006	1.70131230
H	5.28688925	-2.36259691	0.25231051
C	7.16172555	-1.28648421	-0.00508596
O	7.87511485	-2.25036961	0.31994847
O	7.40816668	-0.28364959	-0.70907288
C	4.44743510	-0.22171486	-1.13327034
H	5.39859491	0.01218403	-1.61699453
H	4.06823974	-1.18847258	-1.48338879
H	3.69437158	0.56474612	-1.26219569
C	5.19626881	0.98191206	0.84745518
H	4.40649332	1.71619702	0.65554515
H	5.37720054	0.87784943	1.92336480
H	6.12907817	1.21147273	0.32710455
C	3.43894652	-0.67771604	1.02748054
H	2.72868509	0.13344779	0.83509503
H	3.06472357	-1.62730264	0.63204391
H	3.63998873	-0.77779710	2.09905768
N	4.71597692	-0.33988671	0.33503593
C	-1.27003626	-0.92234455	-1.54659236
H	-1.53301545	-1.71866712	-0.84243396
H	-2.17050354	-0.73313652	-2.14583736
H	-0.47208583	-1.27440438	-2.21297003
C	-2.16064829	3.57161493	0.76678169
H	-2.86457786	3.92464295	-0.00147824
H	-2.76029849	3.20148957	1.60996296
H	-1.58079556	4.43600020	1.11166803

26Me$^-$·Z(2)

C	-4.85806252	-2.91627388	-0.00088494
C	-3.43450262	-3.21099641	-0.00020198
C	-2.47707991	-2.23976489	0.00071805
C	-2.82957308	-0.82244089	0.00077984
C	-4.24948170	-0.50522475	0.00019801
C	-5.18465289	-1.50130836	-0.00052650
C	-1.76154302	0.08686116	0.00154691
H	-0.79185416	-0.41146751	0.00299599
O	-5.72581547	-3.80801807	-0.00165264
C	-1.49260459	1.46070534	0.00090512
C	-0.11261453	1.89187693	0.00246788
C	-1.53994985	3.62297849	-0.00170969
N	-2.32256638	2.59141210	-0.00167339
N	-0.19629460	3.27970304	0.00075939
C	-1.98403177	5.04565626	-0.00406073
H	-1.61458714	5.58186629	-0.89141724
H	-3.07785751	5.06697642	-0.00609506
H	-1.61787833	5.58378160	0.88350615
O	0.97324122	1.27041553	0.00481886
C	0.93988193	4.16365180	0.00116012
H	0.95159969	4.80059214	-0.89446631
H	0.94747640	4.80489850	0.89372944
H	1.83768833	3.53599525	0.00470929
C	4.74812492	-1.81680081	-0.00268909
H	4.61642682	-2.44787914	0.88406039
H	4.61646015	-2.44352396	-0.89252594
C	6.21280290	-1.25732376	-0.00127564
O	7.03843001	-2.18323850	-0.00360997
O	6.36821649	-0.01608770	0.00187466
C	3.66366829	0.04244321	-1.21821446
H	4.62098001	0.56883236	-1.19510830
H	3.60872703	-0.60951897	-2.09679849
H	2.80265680	0.71794330	-1.18431714
C	3.66349362	0.03629467	1.22198590
H	2.80251239	0.71194854	1.19140172
H	3.60843686	-0.62005934	2.09728425
H	4.62080666	0.56278923	1.20164612
C	2.31318472	-1.56570482	-0.00221570
H	1.50083144	-0.83040795	-0.00039606
H	2.26759438	-2.19363178	-0.89762243
H	2.26751531	-2.19815471	0.88999310
N	3.60782342	-0.82566411	-0.00028290
C	-4.73521401	0.91756586	0.00068791
H	-4.36440802	1.46875231	0.87243187
H	-4.36522653	1.46906412	-0.87119418
H	-5.83213242	0.93646939	0.00119708
C	-1.03018844	-2.68699228	0.00139178
H	-0.49223563	-2.32324797	-0.88613684
H	-0.49312752	-2.32354680	0.88958266
H	-0.97998565	-3.78188216	0.00128389
H	-6.24780744	-1.25362910	-0.00078113
H	-3.15787217	-4.26690450	-0.00040867

26Me^{-}·Z(3)

C	4.24745561	-0.29571199	-0.46430009
C	3.84175166	-0.06883337	0.90956329
C	2.77000030	0.72238313	1.25023246
C	1.94244631	1.31666579	0.23647806
C	2.37794497	1.20579438	-1.12740084
C	3.48649478	0.45404496	-1.44232336
C	0.71217354	1.91153180	0.65665750
H	0.69198182	2.35379291	1.65657275
O	5.18976751	-1.06142927	-0.77369792
C	-0.53484744	1.91478354	0.08580539
C	-1.70415663	2.45330208	0.79217146
C	-2.26627216	1.26236611	-1.03929012
N	-0.96913476	1.20206566	-1.04931585
N	-2.77580790	1.99073202	0.02064300
C	-3.16472723	0.58328074	-2.01320723
H	-3.94964676	1.26364526	-2.37072230
H	-2.56749575	0.24444064	-2.86567987
H	-3.64185951	-0.29448905	-1.54391515
O	-1.81617902	3.12166812	1.81559059
C	-4.16406904	2.16685110	0.36871138
H	-4.69171985	2.77162137	-0.38227532
H	-4.66447551	1.19492087	0.46874087
H	-4.18757169	2.69169818	1.32999987
C	-1.42014901	-2.28991578	-0.66283990
H	-1.51314640	-1.27243224	-1.04761042
H	-1.33836760	-2.97826628	-1.51331229
C	-2.69338246	-2.65693103	0.16134627
O	-3.73473236	-2.26615010	-0.40059627
O	-2.53901055	-3.29413256	1.22560673
C	0.27232007	-3.72186514	0.43317547
H	-0.52899402	-4.10580932	1.07091285
H	0.35538162	-4.31669351	-0.48269706
H	1.23916750	-3.69817434	0.94699625
C	-0.14591492	-1.47527204	1.27958867
H	0.85798176	-1.43979091	1.71364312
H	-0.44704291	-0.46855348	0.97995842
H	-0.87499938	-1.91791367	1.96079090
C	0.94831880	-1.77173326	-0.86558049
H	1.91371880	-1.74279264	-0.35077229
H	1.00946882	-2.41091802	-1.75153779
H	0.65090651	-0.75864019	-1.14618449
N	-0.08922280	-2.32857110	0.05319548
H	3.84007588	0.40755604	-2.47472024
H	4.43967963	-0.55334830	1.68452776
C	1.69295376	1.97566539	-2.23244636
H	0.82424285	1.43127998	-2.62059292
H	1.31862313	2.94265652	-1.87160193
H	2.39890977	2.15484669	-3.05319434
C	2.44041867	0.90802664	2.71530357
H	2.45211453	1.97193611	2.99402065
H	1.44261688	0.52533451	2.97390531
H	3.17828606	0.38825891	3.33799325

$35Me^{-}\cdot Z(1)$

C	0.69082677	0.48398750	0.00012395
C	0.13911354	1.81724665	0.00014581
C	-1.22534806	1.99176164	0.00011866
C	-2.14040744	0.90548307	0.00006500
C	-1.59357238	-0.40598770	0.00004693
C	-0.23573602	-0.62554007	0.00007590
H	-1.63054111	3.00828121	0.00012834
H	-2.28212580	-1.25065224	0.00000930
C	-3.53580205	1.18418942	0.00003974
H	-3.80790872	2.24440561	0.00004901
O	1.94850715	0.29242116	0.00013872
C	-4.63051111	0.35672225	0.00000546
C	-6.00176622	0.86796122	-0.00000220
C	-5.90309429	-1.38811132	-0.00007644
N	-4.65396916	-1.05066125	-0.00001622
N	-6.77004215	-0.30570630	-0.00011499
C	-6.41269717	-2.78867995	-0.00013308
H	-7.03216762	-2.98692273	-0.88757727
H	-5.55758515	-3.47088198	-0.00010474
H	-7.03225631	-2.98696063	0.88724074
O	-6.47486169	2.00264326	-0.00004846
C	-8.20862659	-0.32447100	-0.00022925
H	-8.60517599	-0.82566374	-0.89504966
H	-8.60531421	-0.82572712	0.89449422
H	-8.53862999	0.72073336	-0.00021749
C	6.54035192	-1.13517208	0.00029246
H	6.63414430	-1.77109419	0.88865854
H	6.63394052	-1.77195992	-0.88747536
C	7.72743995	-0.11270451	-0.00034814
O	8.81901133	-0.70585062	-0.00010877
O	7.45569283	1.10721075	-0.00100198
C	4.87384233	0.23050279	-1.21789226
H	5.58800422	1.05716588	-1.19898269
H	5.04551978	-0.40103611	-2.09675960
H	3.83078113	0.56364699	-1.17637094
C	4.87415130	0.23177726	1.21746763
H	3.83108158	0.56487878	1.17585042
H	5.04606314	-0.39884030	2.09695085
H	5.58831554	1.05841362	1.19751496
C	4.17386960	-1.74888069	0.00091084
H	3.15367595	-1.34001894	0.00075203
H	4.35919770	-2.35401835	-0.89297579
H	4.35931811	-2.35299557	0.89546431
N	5.12830346	-0.60176429	0.00018972
C	1.10438192	2.96979376	0.00020244
H	1.76393195	2.93159020	0.88004083
H	1.76410420	2.93153191	-0.87950200
H	0.57345268	3.93119919	0.00012461
C	0.34468665	-2.01323114	0.00006723
H	0.98308881	-2.17405448	-0.88267338
H	0.98289149	-2.17415036	0.88293443
H	-0.44926352	-2.77122626	-0.00005340

35Me^{-}·Z(2)

C	5.12632765	-2.00707383	-0.00102345
C	3.83753603	-2.70144806	0.00238309
C	2.67796231	-1.98269393	0.00363942
C	2.64617138	-0.54923019	0.00175872
C	3.90871972	0.13220981	-0.00157213
C	5.09567896	-0.54155460	-0.00292846
H	1.72048320	-2.51441415	0.00621695
H	3.89639778	1.22219011	-0.00302480
C	1.40829186	0.09886679	0.00327250
H	0.53467017	-0.56103342	0.00584430
O	6.20322924	-2.63647178	-0.00223715
C	1.05602418	1.44752921	0.00210767
C	-0.31138237	1.89400790	0.00415608
C	1.14952445	3.60429000	-0.00108065
N	1.91367403	2.55730120	-0.00108850
N	-0.20017328	3.28344487	0.00195291
C	1.61746849	5.01911236	-0.00398235
H	1.26031438	5.56337843	0.88349846
H	2.71152634	5.02180764	-0.00594340
H	1.25714783	5.56077731	-0.89177504
O	-1.40810438	1.29137785	0.00720449
C	-1.32041228	4.18731802	0.00297509
H	-1.31773330	4.82768264	0.89633297
H	-1.32121143	4.82538898	-0.89202572
H	-2.22876491	3.57480776	0.00555262
C	-4.97861255	-2.02383951	-0.00338705
H	-4.80578462	-2.64080530	-0.89308400
H	-4.80637682	-2.64468843	0.88371725
C	-6.47729812	-1.56292993	-0.00287685
O	-7.23909995	-2.54215268	-0.00523075
O	-6.71512345	-0.33498559	-0.00030532
C	-4.01845716	-0.10257523	1.22108507
H	-5.00752934	0.36140524	1.19926676
H	-3.92231730	-0.75341159	2.09701255
H	-3.20257782	0.62695344	1.19129279
C	-4.01776047	-0.09721683	-1.21872802
H	-3.20206204	0.63236199	-1.18523571
H	-3.92094874	-0.74413600	-2.09748266
H	-5.00691111	0.36654041	-1.19550400
C	-2.56526763	-1.61348881	-0.00173618
H	-1.80502153	-0.82353219	0.00037351
H	-2.48083086	-2.24083667	0.89147419
H	-2.48019289	-2.23675896	-0.89773911
N	-3.90580208	-0.95994235	-0.00069602
C	3.86845218	-4.20335650	0.00435017
H	4.41199915	-4.58165941	0.88251587
H	2.85173607	-4.62017952	0.00692584
H	4.40853513	-4.58403078	-0.87492739
C	6.42238006	0.16393223	-0.00641300
H	6.28713198	1.25364626	-0.00770582
H	7.01920494	-0.12391847	0.87152658
H	7.01593293	-0.12645714	-0.88572997

35Me^{-}·Z(3)

C	4.40605400	0.14665062	0.39542890
C	4.13839934	-0.10991694	-1.01464129
C	2.98068205	-0.74005621	-1.39302105
C	1.98257863	-1.15524655	-0.46285240
C	2.26075721	-0.94421684	0.92135043
C	3.40660096	-0.32657888	1.35044813
C	0.74668811	-1.63314820	-0.95928179
H	0.69713789	-1.79157547	-2.04180707
O	5.44377342	0.74002319	0.77100221
C	-0.46891387	-1.83668855	-0.34169415
C	-1.68853055	-2.10008076	-1.10222401
C	-2.09046774	-1.72526710	1.08447715
N	-0.79600945	-1.62298400	1.01156385
N	-2.68998096	-2.00465753	-0.12925668
C	-2.90673742	-1.51114202	2.31130115
H	-3.53710408	-2.38607899	2.52544262
H	-2.23312298	-1.33740866	3.15611633
H	-3.56521148	-0.63897850	2.17089241
O	-1.89085632	-2.30792364	-2.29949202
C	-4.10756736	-2.01509555	-0.41176896
H	-4.60658280	-2.85007255	0.09980828
H	-4.56276590	-1.06327970	-0.10583714
H	-4.21378780	-2.14543686	-1.49397711
C	-2.02124453	1.71970813	0.69366304
H	-1.83659897	0.66384172	0.90138440
H	-1.91365531	2.27650480	1.63269077
C	-3.47574417	1.90359780	0.16289627
O	-4.29144809	1.23842698	0.82975409
O	-3.65700018	2.67495993	-0.80344980
C	-0.90640988	3.63956284	-0.39121976
H	-1.87392522	3.89760549	-0.83164211
H	-0.79112139	4.11326422	0.58947022
H	-0.06801236	3.91847685	-1.03879629
C	-0.99198354	1.48621974	-1.53240479
H	-0.11912807	1.76673475	-2.13054793
H	-0.98846177	0.40593068	-1.36832201
H	-1.92457512	1.81018025	-1.99820333
C	0.39440984	1.75190458	0.44924487
H	1.23643788	1.97548452	-0.21386183
H	0.49922666	2.29624388	1.39253220
H	0.36063984	0.67889739	0.64418345
N	-0.88442756	2.16308470	-0.20260976
H	2.79367888	-0.91532040	-2.45759457
H	1.51792316	-1.27538360	1.64816562
C	3.69475404	-0.09730748	2.80774712
H	4.63413771	-0.58752332	3.10230590
H	3.83845914	0.97389575	3.01404961
H	2.87901175	-0.47979512	3.43619429
C	5.17494610	0.34382212	-2.00385768
H	5.34763990	1.42736033	-1.92223565
H	6.14543625	-0.13207711	-1.79910582
H	4.87187214	0.10619140	-3.03325006

$35Bu^{-}\cdot Z(1)$

C	0.48396364	0.29355584	0.39474969
C	0.00803214	1.66679047	0.30336142
C	-1.34087365	1.89565130	0.14043989
C	-2.30040223	0.85887234	0.06618776
C	-1.83448178	-0.47263824	0.17866358
C	-0.50258880	-0.77951246	0.34348591
C	-3.67247406	1.20038334	-0.10717130
H	-3.89295901	2.27025952	-0.17581295
O	1.72653839	0.03882180	0.51904140
C	-4.79407371	0.41786442	-0.20626894
C	-6.13425839	0.97989381	-0.38517809
C	-6.13035333	-1.27654446	-0.29548267
N	-4.87677747	-0.98673499	-0.16000482
N	-6.94539668	-0.16333565	-0.43504658
C	-6.69315413	-2.65635839	-0.30709772
H	-7.21222256	-2.86611545	-1.25415650
H	-5.87239778	-3.36860593	-0.18231938
H	-7.41974783	-2.79649265	0.50703987
O	-6.55525249	2.13037991	-0.47947038
C	-8.37385780	-0.12901746	-0.60461322
H	-8.68306612	-0.64898414	-1.52274118
H	-8.89220570	-0.58110828	0.25321373
H	-8.65870618	0.92672489	-0.67856181
C	6.43927907	-1.17050064	-0.08612403
H	6.83500273	-1.25620093	0.93279672
H	6.39206741	-2.17549837	-0.52178687
C	7.43747580	-0.30959189	-0.93328451
O	8.56673216	-0.82602598	-0.94169791
O	7.00547089	0.73352159	-1.46917950
C	4.35344372	-0.61225188	-1.29475989
H	4.92329375	0.09972618	-1.89594408
H	4.38547782	-1.60929280	-1.74678302
H	3.31644009	-0.30032141	-1.13370047
C	4.96155009	0.65537115	0.69312967
H	3.90817886	0.92773594	0.80558243
H	5.44424359	0.57738178	1.67320160
H	5.51143598	1.34771311	0.05156898
C	4.25159788	-1.65345343	0.89359578
H	3.22139435	-1.28927853	0.97682574
H	4.27568791	-2.63804938	0.41553842
H	4.73177269	-1.70869414	1.87569383
N	5.01356038	-0.69213941	0.04580398
H	-1.71444324	2.91665478	0.06341359
H	-2.58396872	-1.25639320	0.13016762
C	1.01238140	2.82559685	0.39289038
C	-0.04659861	-2.23992546	0.48480908
C	0.62968750	-2.44103496	1.85565897
H	1.03969503	-3.45987025	1.93707223
H	1.43412257	-1.71605125	2.00844388
H	-0.10655972	-2.30528864	2.66004575
C	-1.22315761	-3.22525116	0.41087484
H	-1.95878185	-3.04686511	1.20670029
H	-1.74591138	-3.16760670	-0.55351330
H	-0.84174074	-4.25048702	0.52757301
C	1.75194341	2.77111928	1.74468513
H	2.21778760	1.79294496	1.89503418
H	2.52627683	3.55228286	1.78969758
H	1.04455799	2.94336656	2.56810851
C	0.32851614	4.19827816	0.30668113
H	-0.19100954	4.33894217	-0.65140692
H	-0.39637266	4.34756673	1.11909325
H	1.09081587	4.98615526	0.39014680
C	0.92354775	-2.60890638	-0.65446989
H	1.78373312	-1.93645723	-0.66879283
H	1.27764343	-3.64480382	-0.53473794
H	0.41187980	-2.53406314	-1.62396754
C	2.01762100	2.74698453	-0.77233503
H	2.76299760	3.55260998	-0.69296979
H	2.53739870	1.78655652	-0.77740625
H	1.49398183	2.85955088	-1.73174172

$35Bu^{-} \cdot Z(2)$

C	4.14059139	-1.23344786	0.00079348
C	2.90946745	-2.04336134	-0.00186223
C	1.70025995	-1.40554134	-0.00317791
C	1.55957038	0.01476063	-0.00212425
C	2.75489061	0.79577724	0.00044198
C	4.00149901	0.23491806	0.00189074
H	0.77590018	-1.98475845	-0.00515899
H	2.62539041	1.87419674	0.00121007
C	0.27269716	0.56676354	-0.00361090
H	-0.54781938	-0.15784657	-0.00555034
O	5.26782010	-1.77559643	0.00204915
C	-0.17535602	1.88320799	-0.00306285
C	-1.57321206	2.23211516	-0.00469368
C	-0.23566806	4.04088324	-0.00098838
N	0.60115817	3.05179153	-0.00079249
N	-1.55990057	3.62535837	-0.00330368
C	0.13152050	5.48488758	0.00098152
H	-0.26638120	5.99960612	0.88854871
H	1.22259186	5.56437286	0.00267650
H	-0.26374937	6.00136690	-0.88673950
O	-2.62166690	1.55237646	-0.00681276
C	-2.74104247	4.44866517	-0.00418645
H	-2.78571567	5.08516209	0.89065649
H	-2.78301295	5.08711037	-0.89776962
H	-3.60425789	3.77410038	-0.00623226
C	-5.84516044	-2.11832731	0.00337159
H	-5.61325809	-2.71810564	-0.88459678
H	-5.61042641	-2.71573125	0.89219427
C	-7.38224382	-1.80789999	0.00539733
O	-8.04332268	-2.85749073	0.00772275
O	-7.74007686	-0.60930995	0.00446739
C	-5.07956505	-0.10692657	1.21954335
H	-6.11079483	0.25373397	1.19909175
H	-4.91492199	-0.74130605	2.09731056
H	-4.34306411	0.70234591	1.18530209
C	-5.08352502	-0.11014301	-1.22057801
H	-4.34685999	0.69915134	-1.19092990
H	-4.92181389	-0.74687179	-2.09718721
H	-6.11466994	0.25063280	-1.19770671
C	-3.48459006	-1.46888094	-0.00131999
H	-2.80789184	-0.60655271	-0.00360798
H	-3.33515813	-2.08110664	0.89380709
H	-3.33811510	-2.08354866	-0.89526043
N	-4.88358999	-0.95265760	0.00027502
C	3.02326079	-3.57369010	-0.00309893
C	5.26804887	1.10170197	0.00463863
C	3.77570558	-4.04438196	-1.26317842
H	3.23266375	-3.73619679	-2.16833318
H	4.78181039	-3.61551656	-1.29312045
H	3.85390715	-5.14278420	-1.26858685
C	1.64586724	-4.25318796	-0.00569716
H	1.78098888	-5.34415485	-0.00633967
H	1.05757752	-3.99154067	0.88543514
H	1.06024775	-3.99017499	-0.89818530
C	3.77211112	-4.04670736	1.25824936
H	3.22648215	-3.74020539	2.16242076
H	3.85029355	-5.14511753	1.26183976
H	4.77812140	-3.61788777	1.29185138
C	6.10774592	0.81126201	-1.25483866
H	6.40634307	-0.24097456	-1.28674328
H	5.52798446	1.04310568	-2.15996808
H	7.01170126	1.44024755	-1.25811641
C	4.93310794	2.60066894	0.00523805
H	4.35540411	2.89186528	0.89339268
H	5.86864433	3.17879905	0.00721917
H	4.35834028	2.89322372	-0.88437295
C	6.10357775	0.80935383	1.26644474
H	7.00749028	1.43836754	1.27367427
H	5.52081628	1.03979555	2.17000400
H	6.40215723	-0.24290139	1.29776932

35Bu^{-}·Z(3)

C	-3.15424750	-0.38205521	-0.02566486
C	-2.84963467	0.73594232	0.86816764
C	-1.70496586	0.69372110	1.63331664
C	-0.77283849	-0.36901513	1.57203311
C	-1.10782069	-1.48755119	0.77071369
C	-2.25375211	-1.53802906	0.00994530
C	0.51853857	-0.16448587	2.14758187
H	0.60594524	0.65126999	2.87282404
O	-4.14671124	-0.34639926	-0.80432579
C	1.72189141	-0.71900441	1.80857234
C	2.99872564	-0.16031381	2.27537467
C	3.25484455	-1.66282535	0.61590064
N	1.96807857	-1.66391031	0.79288974
N	3.93372701	-0.79972938	1.45774822
C	3.97657787	-2.45342472	-0.41666212
H	4.78647718	-3.04620764	0.03155969
H	3.26451976	-3.12304884	-0.90840486
H	4.41158372	-1.76088506	-1.15327876
O	3.26412343	0.68406912	3.12562827
C	5.32855809	-0.43063064	1.37041291
H	5.97797663	-1.28932104	1.59028066
H	5.54851003	-0.03627915	0.36906138
H	5.49897454	0.34809215	2.12126384
C	2.16333239	0.66302402	-1.85958507
H	2.08039749	-0.10938773	-1.08812931
H	2.04709034	0.18119470	-2.83786704
C	3.57539270	1.32337874	-1.79452477
O	4.48002173	0.46560280	-1.79349629
O	3.64774857	2.57005462	-1.77295422
C	0.88836184	2.57538398	-2.77107912
H	1.81243200	3.15907195	-2.74006994
H	0.79800769	2.04480474	-3.72487745
H	0.00101091	3.19638443	-2.60739456
C	1.03489724	2.25659528	-0.35667675
H	0.10362281	2.81230112	-0.20704834
H	1.13597070	1.49401724	0.41919875
H	1.90841119	2.91095316	-0.37241975
C	-0.26576655	0.71319682	-1.71123445
H	-1.15024996	1.33495413	-1.54220425
H	-0.33032876	0.22271412	-2.68652948
H	-0.19988549	-0.03651011	-0.92085059
N	0.95957453	1.56455219	-1.68016467
H	-1.44981212	1.53313744	2.28135101
H	-0.39353405	-2.30489308	0.75081285
C	-2.56910202	-2.75671829	-0.86769835
C	-3.78443531	1.95283451	0.88598990
C	-1.54952069	-3.88773953	-0.67008126
H	-0.53145200	-3.58045161	-0.94603968
H	-1.52863265	-4.23764788	0.37147595
H	-1.82767586	-4.74026982	-1.30672617
C	-2.53081631	-2.34839219	-2.35406589
H	-2.79242393	-3.20736127	-2.99149089
H	-3.23640130	-1.53342037	-2.54509571
H	-1.51627240	-2.02444484	-2.63060837
C	-3.96197230	-3.31346070	-0.51678513
H	-4.73072132	-2.55016293	-0.67155875
H	-4.18998028	-4.18813001	-1.14603581
H	-3.98709438	-3.63428095	0.53469641
C	-3.78968528	2.61591431	-0.50595412
H	-4.09443080	1.89344607	-1.27001126
H	-4.48326208	3.47098044	-0.52169092
H	-2.78525260	2.99838547	-0.74782485
C	-3.34057474	3.01338503	1.90338058
H	-3.31670563	2.61232724	2.92637986
H	-2.34598787	3.42012252	1.67043268
H	-4.05238541	3.85126833	1.88804440
C	-5.21442187	1.51861110	1.26032540
H	-5.58651004	0.77687356	0.54721839
H	-5.22361044	1.07791606	2.26764335
H	-5.88883683	2.38943525	1.26156794

$Phe^{-} \cdot Z(1)$

C	1.934820	-0.994475	-0.016419
C	1.533144	-2.379620	0.007121
C	0.209259	-2.742032	0.013184
C	-0.841731	-1.782956	-0.002178
C	-0.460824	-0.408981	-0.027698
C	0.857960	-0.032168	-0.034727
H	-0.057070	-3.802301	0.031470
H	-1.246710	0.344633	-0.044583
C	-2.185259	-2.238178	0.009182
H	-2.320879	-3.324248	0.022811
O	3.151681	-0.643670	-0.021545
C	-3.378636	-1.553215	0.009962
C	-4.675314	-2.226206	0.026781
C	-4.856806	0.023774	-0.002910
N	-3.572205	-0.170244	-0.000216
N	-5.588577	-1.160319	0.001606
O	-5.001724	-3.410472	0.072851
C	-7.004478	-1.372522	0.180098
H	-7.419629	-0.642650	0.885159
H	-7.554732	-1.310760	-0.770033
H	-7.126125	-2.386294	0.578526
H	2.320604	-3.135077	0.020068
H	1.135427	1.022830	-0.055901
C	7.924963	0.080779	-0.020662
H	8.288516	-0.450590	0.866987
H	8.288485	-0.448763	-0.909410
C	8.543065	1.520643	-0.019105
O	9.784045	1.465854	-0.019254
O	7.763603	2.497482	-0.017883
C	5.828471	0.578990	-1.238231
H	6.107559	1.635199	-1.217215
H	6.260247	0.088472	-2.117848
H	4.744852	0.422210	-1.198147
C	5.828465	0.577009	1.197655
H	4.744854	0.420314	1.157176
H	6.260100	0.084961	2.076496
H	6.107683	1.633222	1.178396
C	6.067890	-1.508643	-0.021914
H	4.971872	-1.590891	-0.021261
H	6.499699	-1.969138	-0.916997
H	6.500808	-1.970764	0.871798
N	6.421659	-0.059207	-0.020787
C	-5.450855	1.372052	-0.008403
C	-6.676898	1.665321	-0.619694
C	-4.728339	2.416218	0.588746
C	-7.172483	2.968355	-0.624864
H	-7.240074	0.882392	-1.123177
C	-5.224982	3.714766	0.582235
H	-3.768733	2.185952	1.048774
C	-6.452556	3.997095	-0.020717
H	-8.124103	3.180756	-1.113134
H	-4.650850	4.513830	1.052051
H	-6.842037	5.015515	-0.024258

$Phe^{-}\cdot Z(2)$

C	6.010778	2.160073	-0.044503
C	6.022832	3.479026	-0.505686
C	4.813516	4.127831	-0.745587
C	3.603435	3.472360	-0.520138
C	3.580863	2.152560	-0.046547
C	4.806626	1.505011	0.183496
H	6.950460	1.638523	0.141437
H	6.968290	3.993321	-0.681392
H	4.808193	5.151868	-1.121253
H	2.673684	3.990075	-0.744703
H	4.786835	0.475414	0.537097
C	2.342039	1.393583	0.181699
C	0.185731	0.900940	0.646132
C	0.985934	-0.280656	0.377131
N	2.298511	0.094019	0.107700
C	0.761345	3.307065	0.844564
H	1.553234	3.766315	1.449158
H	0.573907	3.933552	-0.040389
H	-0.167558	3.252625	1.424588
N	1.105828	1.953094	0.490291
O	-0.996438	1.067949	0.961152
C	0.459685	-1.566540	0.405390
H	-0.607605	-1.573914	0.649659
C	1.045495	-2.822562	0.184547
C	0.234305	-3.998501	0.295998
C	2.426217	-3.023352	-0.150800
C	0.730301	-5.255568	0.100418
H	-0.822084	-3.869367	0.549366
C	2.939696	-4.272345	-0.350623
H	3.061770	-2.143623	-0.246125
C	2.130239	-5.482460	-0.238147
H	0.092764	-6.136657	0.191661
H	3.991764	-4.410969	-0.606777
O	2.601220	-6.623221	-0.418954
C	-5.569397	1.124507	-0.907915
H	-5.792165	2.198350	-0.941907
H	-5.489670	0.755915	-1.939046
C	-6.766114	0.394496	-0.203252
O	-7.823830	0.604845	-0.812694
O	-6.523897	-0.269483	0.826282
C	-3.761976	-0.441429	-0.268117
H	-4.490097	-0.985601	0.340700
H	-3.761346	-0.817733	-1.296736
H	-2.751928	-0.475707	0.150795
C	-4.184410	1.521549	1.102245
H	-3.170153	1.389619	1.493568
H	-4.450830	2.581059	1.065281
H	-4.932075	0.965443	1.673701
N	-4.194729	0.989516	-0.296357
C	-3.222516	1.766974	-1.119064
H	-3.557869	2.808520	-1.161753
H	-2.239271	1.694999	-0.642250
H	-3.199715	1.345472	-2.129841

Phe$^-$·Z(3)

C	-5.343456	-0.717167	-1.010568
C	-5.440909	-0.160674	0.332680
C	-4.376632	-0.159673	1.190924
C	-3.098515	-0.693814	0.833649
C	-2.989482	-1.277031	-0.470979
C	-4.042133	-1.289314	-1.342733
C	-2.018925	-0.554056	1.730819
H	-2.251732	-0.090047	2.694537
O	-6.295817	-0.705718	-1.815956
C	-0.670868	-0.828768	1.584934
C	0.323480	-0.431146	2.575825
C	1.248993	-1.317582	0.722139
N	-0.028243	-1.384773	0.473610
N	1.537845	-0.751731	1.952400
O	0.226945	0.078937	3.692434
C	2.822969	-0.506848	2.572347
H	3.505486	-1.338942	2.365518
H	3.260270	0.432944	2.209483
H	2.646226	-0.430901	3.650620
C	1.198907	1.784390	-0.350041
H	0.867127	1.392931	0.616798
H	1.500700	0.939681	-0.977340
C	2.431325	2.716102	-0.130747
O	3.372590	2.097798	0.395454
O	2.344651	3.911641	-0.490255
C	0.310810	2.912599	-2.352401
H	1.079823	3.679066	-2.223511
H	0.690458	2.081379	-2.955404
H	-0.600607	3.315404	-2.807281
C	-0.570417	3.480200	-0.144616
H	-1.480336	3.870957	-0.612828
H	-0.811297	3.053490	0.834184
H	0.207224	4.243306	-0.055308
C	-1.070331	1.320161	-1.134991
H	-1.938418	1.729142	-1.661238
H	-0.652729	0.471352	-1.682282
H	-1.365009	0.981520	-0.140903
N	-0.030808	2.384043	-1.001683
H	-3.944257	-1.739759	-2.332319
H	-6.403446	0.261117	0.626011
H	-4.489854	0.276682	2.187606
H	-2.032076	-1.710657	-0.758860
C	2.270076	-1.778680	-0.236194
C	2.023036	-2.949352	-0.966809
C	3.441315	-1.047676	-0.481632
C	2.939745	-3.393865	-1.914603
H	1.100398	-3.497706	-0.781450
C	4.353797	-1.498471	-1.434713
H	3.615069	-0.094829	0.023118
C	4.112624	-2.672016	-2.147739
H	2.739731	-4.308625	-2.473311
H	5.254066	-0.914444	-1.627570
H	4.832227	-3.021003	-2.889308

rKFP^{-}·Z(1)

C	-2.440307	0.406196	-0.463384
C	-2.498390	-1.038038	-0.411575
C	-1.361665	-1.798797	-0.310075
C	-0.062162	-1.219345	-0.253233
C	0.013724	0.205791	-0.311701
C	-1.112551	0.978712	-0.412071
H	-1.444850	-2.888157	-0.273215
H	0.997737	0.670476	-0.273513
C	1.061780	-2.071677	-0.145285
H	0.847247	-3.144422	-0.112694
O	-3.479796	1.120173	-0.539301
C	2.411899	-1.796543	-0.070571
C	3.421978	-2.847712	0.040076
C	4.318071	-0.775520	0.006559
N	3.032828	-0.553286	-0.085151
N	4.625890	-2.128748	0.085531
O	3.346524	-4.073082	0.089217
C	5.902144	-2.788424	0.194041
H	6.539814	-2.576667	-0.675327
H	6.427150	-2.500612	1.115361
H	5.689345	-3.862978	0.226326
H	-3.480656	-1.511147	-0.462138
H	-1.042215	2.066369	-0.456821
C	-8.069347	0.216362	0.908411
H	-8.108555	-0.863898	1.092384
H	-8.165470	0.731659	1.871496
C	-9.295191	0.610364	0.014861
O	-10.361798	0.308082	0.573985
O	-9.069523	1.150639	-1.089466
C	-6.503559	1.985458	0.170331
H	-7.246090	2.290306	-0.571161
H	-6.686235	2.494939	1.123182
H	-5.473721	2.147849	-0.163765
C	-6.421595	-0.206678	-0.889316
H	-5.397568	0.032990	-1.196793
H	-6.531431	-1.280194	-0.698689
H	-7.173190	0.131045	-1.606702
C	-5.680961	0.068153	1.406694
H	-4.683997	0.315729	1.023952
H	-5.880793	0.589206	2.348675
H	-5.790625	-1.011237	1.553436
N	-6.681192	0.516303	0.395649
C	5.245056	0.338102	0.016900
H	4.698178	1.280304	-0.061374
C	6.590421	0.344014	0.104974
C	7.413476	1.537302	0.104616
C	8.783511	1.682729	0.191792
C	8.015644	3.666558	0.044431
H	5.976333	3.106383	-0.069986
H	9.521010	0.891323	0.280294
H	7.912792	4.744713	-0.014264
H	7.153806	-0.582303	0.184318
N	6.942360	2.830425	0.010655
N	9.146812	3.006704	0.153697

rKFP^{-}·Z(2)

C	1.007286	6.204620	0.014010
C	-0.388720	5.767696	-0.000525
C	-0.720285	4.448747	-0.010068
C	0.267911	3.402171	-0.006573
C	1.648932	3.813042	0.007712
C	1.999131	5.127344	0.017415
H	-1.774296	4.156296	-0.020802
H	2.411291	3.035371	0.010558
C	-0.151801	2.076226	-0.016826
H	-1.235348	1.922495	-0.027163
O	1.328425	7.403440	0.022838
C	0.562563	0.870279	-0.016449
C	-0.083386	-0.417941	-0.027543
C	2.158932	-0.588876	-0.009850
N	1.934807	0.703059	-0.005817
N	0.975762	-1.321069	-0.022893
O	-1.284424	-0.760518	-0.038687
C	0.773921	-2.748468	-0.029954
H	1.211196	-3.208878	-0.926008
H	1.199288	-3.215908	0.868169
H	-0.309052	-2.911465	-0.037832
H	-1.151217	6.547669	-0.003325
H	3.047005	5.431321	0.028233
C	-6.166826	-1.007620	0.001508
H	-6.481553	-0.434636	0.881677
H	-6.494064	-0.468184	-0.895143
C	-6.885674	-2.400935	0.032763
O	-8.117875	-2.259993	0.038597
O	-6.173269	-3.428843	0.047151
C	-4.129915	-1.693862	-1.219671
H	-4.492888	-2.723629	-1.174012
H	-4.530990	-1.187571	-2.104414
H	-3.037659	-1.623952	-1.198205
C	-4.112510	-1.646034	1.219687
H	-3.020694	-1.576643	1.180064
H	-4.501518	-1.105887	2.089666
H	-4.474724	-2.677005	1.219350
C	-4.184438	0.427156	-0.040524
H	-3.088264	0.418672	-0.048289
H	-4.578529	0.907793	-0.941747
H	-4.565551	0.942603	0.846952
N	-4.655615	-0.987018	-0.009563
C	3.510812	-1.099478	-0.001182
H	4.221195	-0.269427	0.009134
C	3.965191	-2.370288	-0.004192
C	5.365830	-2.745133	0.005555
C	5.963981	-3.989458	0.003488
C	7.575201	-2.592397	0.025210
H	6.360657	-0.856657	0.024931
H	5.466820	-4.954250	-0.006283
H	8.556028	-2.129506	0.036262
H	3.280995	-3.215074	-0.014553
N	6.425759	-1.862318	0.019636
N	7.333886	-3.883652	0.015729

rKFP^{-}·Z(3)

C	5.397230	-1.867178	1.012920
C	5.726074	-0.738557	0.150209
C	4.815359	-0.210579	-0.720925
C	3.480606	-0.714908	-0.840365
C	3.145630	-1.845418	-0.024075
C	4.042791	-2.389725	0.850317
C	2.566151	-0.036984	-1.667608
H	2.967405	0.813891	-2.228014
O	6.207114	-2.349681	1.827909
C	1.197417	-0.180325	-1.832629
C	0.394339	0.822823	-2.520930
C	-0.864887	-0.711525	-1.453768
N	0.357963	-1.112897	-1.220360
N	-0.919788	0.442538	-2.219078
O	0.699569	1.814179	-3.186265
C	-2.075562	1.178458	-2.688993
H	-2.845564	0.483426	-3.043934
H	-2.488270	1.815272	-1.895354
H	-1.732721	1.808828	-3.516116
C	-0.810992	1.495968	1.211325
H	-0.757028	0.896778	0.301912
H	-1.226129	0.867652	2.008211
C	-1.766580	2.704860	0.966710
O	-2.835543	2.337252	0.441007
O	-1.387179	3.840541	1.318797
C	0.684624	2.509037	2.897765
H	0.124370	3.441660	2.786800
H	0.221840	1.872674	3.659405
H	1.737005	2.682210	3.147687
C	1.264947	2.630452	0.530881
H	2.315024	2.781331	0.801236
H	1.204190	2.093875	-0.419013
H	0.720731	3.575570	0.478870
C	1.353870	0.494420	1.700511
H	2.417829	0.685967	1.872196
H	0.932897	-0.074334	2.534882
H	1.224281	-0.066117	0.772536
N	0.623743	1.793334	1.592381
H	3.774399	-3.250741	1.465088
H	6.735189	-0.331203	0.229306
H	5.099669	0.640335	-1.346873
H	2.142236	-2.261559	-0.111983
C	-2.015908	-1.393813	-0.875899
C	-3.142033	-0.799418	-0.437076
H	-3.249023	0.287142	-0.474805
H	-1.839895	-2.460677	-0.720144
C	-4.244650	-1.486427	0.203315
C	-5.375984	-0.983453	0.812324
C	-5.523162	-3.103950	1.007473
H	-3.711580	-3.544002	0.001052
H	-5.641807	0.063023	0.919395
H	-5.853240	-4.110327	1.242442
N	-6.162179	-1.995149	1.308945
N	-4.362476	-2.855190	0.343078
